# Enzyme Dynamics Determine the Potency and Selectivity of Inhibitors Targeting Disease-Transmitting Mosquitoes

**DOI:** 10.1021/acsinfecdis.4c00531

**Published:** 2024-09-18

**Authors:** Rashmi Kumari, Cecilia Lindgren, Rajendra Kumar, Nina Forsgren, C. David Andersson, Fredrik Ekström, Anna Linusson

**Affiliations:** † Department of Chemistry, Umeå University, Umeå SE-90187, Sweden; ‡ CBRN Defense and Security, Swedish Defense Research Agency, Umeå SE-90621, Sweden

**Keywords:** acetylcholinesterase, molecular dynamics, inhibitors, vector control, mosquitoes

## Abstract

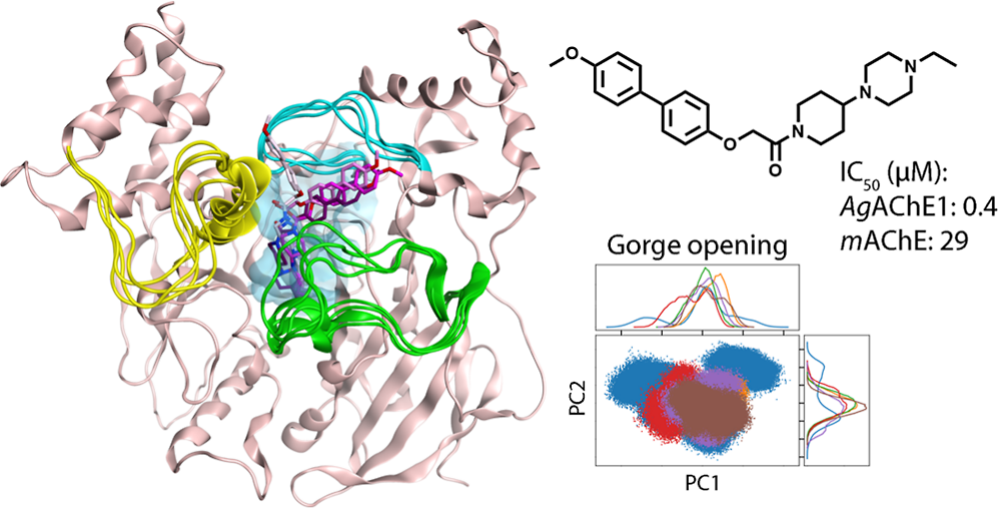

Vector control of mosquitoes with insecticides is an important tool for preventing the spread of mosquito-borne diseases including malaria, dengue, chikungunya, and Zika. Development of active ingredients for insecticides are urgently needed because existing agents exhibit off-target toxicity and are subject to increasing resistance. We therefore seek to develop noncovalent inhibitors of the validated insecticidal target acetylcholinesterase 1 (AChE1) from mosquitoes. Here we use molecular dynamics simulations to identify structural properties essential for the potency of reversible inhibitors targeting AChE1 from *Anopheles gambiae* (*Ag*AChE1), the malaria-transmitting mosquito, and for selectivity relative to the vertebrate *Mus musculus* AChE (*m*AChE). We show that the collective motions of apo *Ag*AChE1 and *m*AChE differ, with *Ag*AChE1 exhibiting less dynamic movement. Opening and closing of the gorge, which regulates access to the catalytic triad, is enabled by different mechanisms in the two species, which could be linked to their differing amino acid sequences. Inhibitor binding reduced the overall magnitude of dynamics of AChE. In particular, more potent inhibitors reduced the flexibility of the Ω loop at the entrance of the gorge. The selectivity of inhibitors for *Ag*AChE1 over *m*AChE derives from the positioning of the α-helix lining the binding gorge. Our findings emphasize the need to consider dynamics when developing inhibitors targeting this enzyme and highlight factors needed to create potent and selective *Ag*AChE1 inhibitors that could serve as active ingredients to combat disease-transmitting mosquitoes.

Vector-borne diseases are caused by parasites, viruses, or bacteria and are most commonly transmitted *via* bloodsucking insect vectors. Mosquitoes are the most well-known vectors, but other examples include fleas, blackflies, and ticks. Over 17% of all infectious diseases around the world are vector-borne diseases that mainly affect tropical and subtropical areas.^1^ The most devastating of these diseases is malaria, which is caused by *Plasmodium* protozoan parasites transmitted *via Anopheles* mosquitoes, with *Anopheles gambiae* being the main vector. In the year 2021 the World Health Organization (WHO) estimated the number of global malaria cases to be 247 million with 619,000 deaths. *Anopheles* mosquitoes also transmit other parasitic infections including lymphatic filariasis.^2^ Climate change and greater global connectivity due to trade and travel have increased the incidence of mosquito-borne diseases and spread them to new areas of the world.^3^

The most effective strategy to reduce the spread of mosquito-borne diseases involves vector control using insecticides, which may be sprayed indoors or used to treat bed nets. The active ingredients of the most commonly used insecticides can be divided into four distinct chemical classes and act by disrupting the nervous system of the insect vectors, causing paralysis and death. Organophosphates and carbamates target the enzyme acetylcholinesterase (AChE), while pyrethroids and organochlorines target voltage-gated ion channels. Despite the success of vector control at reducing the spread of mosquito-borne diseases,^4^ the heavy usage of a few insecticides has driven the development of resistance to all four major classes of insecticides recommended by WHO.^5^ Documented mechanisms of resistance include increased rates of metabolism, reduced uptake of the insecticide, changes in mosquitoes’ behavior, and genetic mutations of the molecular target that reduce the insecticides’ potency.^6,7^

The well-established insecticidal target AChE is an essential enzyme found in insects as well as other animals and humans. It plays a key role in terminating nerve signaling in the synaptic cleft by catalyzing the rapid hydrolysis of the neurotransmitter acetylcholine.^8^ Inhibition of this enzyme causes acetylcholine accumulation, resulting in overstimulation of the nervous system and eventually death due to acute cholinergic crisis. While vertebrates have only one gene coding for AChE, most insects have two: *ace-1* and *ace-2*.^9^ Most insects thus carry two isoforms of AChE, but in mosquitoes AChE1 is responsible for terminating nerve signaling and the function of AChE2 is not fully understood.^10^ The catalytic site of AChE is located at the bottom of a 20 Å deep gorge, where the catalytic triad of Ser, His, and Glu residues is found. The amino acid residues flanking this gorge can be assigned to five subdomains: the Ω-loop and subdomains S1, S2, S3, and S4 (Figure 1). The entrance of the gorge, called the peripheral site, is defined by three loops: the Ω loop, loop 1 (part of S2), and loop 2 (part of S3). Both organophosphates and carbamates abolish the enzymatic activity of AChE by reacting with the catalytic serine, forming a covalent bond. Organophosphates and carbamates have a broad inhibition profile and thus often exhibit limited selectivity between AChE variants from different species. Consequently, they often have significant off-target toxicity and may cause significant harm to humans, animals, and beneficial insects, limiting their practical use as insecticides.^11,12^ New insecticides that avoid established resistance mechanisms while also having more favorable selectivity profiles are thus urgently needed to enable more effective vector control.

**Figure 1:**
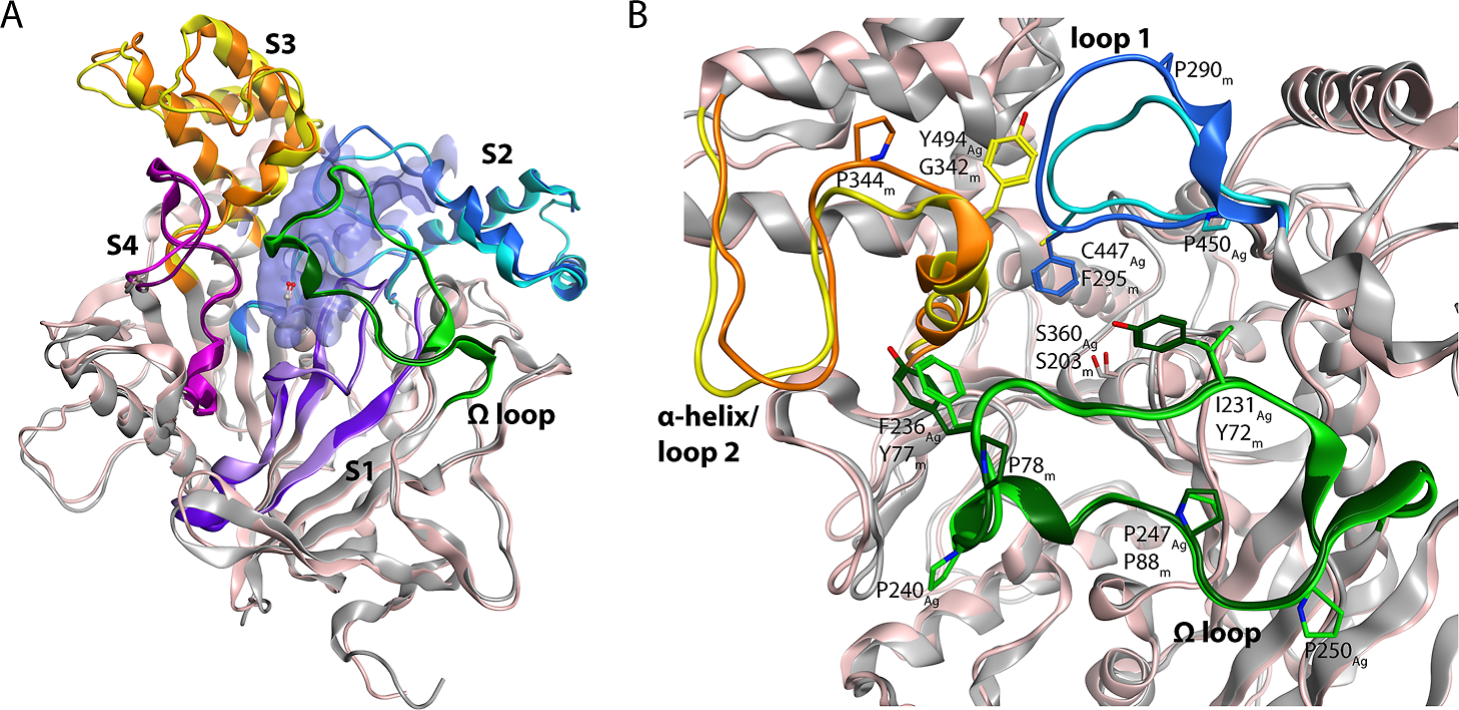
(A) The tertiary structure of *Ag*AChE1 (pink ribbons; light colors) superpositioned to *m*AChE (gray ribbons; dark colors). The five subdomains Ω-loop, S1, S2, S3, and S4 (green, purple, blue, yellow, and magenta, respectively) and the active site gorge (surface displayed) with its catalytic serine at the bottom are highlighted. (B) Close up of the entrance of the active site gorge viewed from above with amino acid residue differences shown. Residues are displayed as sticks and colored according to the ribbon with oxygen in red, nitrogen in blue, and sulfur in yellow. The figures are based on protein coordinates of previously published crystal structures (PDB codes 5X61 and 1J06, for *Ag*AChE1 and *m*AChE, respectively). The highlighted subdomains have the following sequences: *Ag*AChE1; the Ω loop (Cys228_Ag_-Cys255_Ag_), S1 (Trp255_Ag_-Val311_Ag_), S2 (Gln385_Ag_-Val454_Ag_), S3 (Asn483_Ag_-Phe560_Ag_), and S4 (Lys588_Ag_-Glu610_Ag_), and *m*AChE; the Ω loop (Cys69_m_-Cys96_m_), S1 (Trp117_m_-Val153_m_), S2 (Gln228_m_-Val302_m_), S3 (Val331_m_-Asn406_m_), and S4 (Ser435_m_-Leu457_m_).

Several different strategies have been used in efforts to discover and develop more potent and selective AChE1 inhibitors for mosquito control, including creating new covalent inhibitors targeting the conserved catalytic serine^13−15^ and the AChE1-specific residue Cys447_Ag_.^16,17^ However, the efficacy of the latter strategy was questioned by a recent study.^18^ A different approach is to focus on noncovalent inhibitors that could potentially target any site(s) or residue(s) within the AChE1 protein rather than being limited to the catalytic serine.^19−25^ We previously performed a high throughput screen of a library containing 17 500 compounds to identify inhibitors of *A. gambiae* AChE1 (*Ag*AChE1) and *Aedes aegypti* AChE1 (*Aa*AChE1).^20^ By combining our results with those of an earlier screen targeting *Homo sapiens* AChE, (*h*AChE)^26^ we identified 163 inhibitors that were selective for AChE1, 74 selective for *h*AChE, and 37 that were nonselective. However, we also found it impossible to establish a general structure–activity relationship (SAR) for selectivity toward AChE1 over *h*AChE based solely on inhibition kinetics and X-ray crystallographic structures,^19−22^ suggesting that this SAR instead depends on other factors. In an earlier study by our group, molecular dynamics (MD) simulations provided valuable insights into the potency and selectivity of a set of phenoxyacetamide-based AChE inhibitors that could not be obtained by exploring static structures of AChEs in complex with inhibitors.^22^

The impact of the dynamics of AChE on its natural function and their importance in drug development targeting the enzyme are widely acknowledged.^27,28^ Although the catalytic site of AChE is located at the bottom of the narrow gorge, the hydrolysis of the neurotransmitter ACh is surprisingly fast, with a turnover number on the order of ∼10,000 s^–1^.^29^ Several computational studies have examined the trafficking of the substrate and products to and from the active site as well as the dynamics of AChE, as reviewed by Xu *et al.*(27) and De Boer *et al.*(28) MD simulations of AChE•inhibitor complexes have also been used to complement inhibitor docking studies by providing data on the stability of predicted complexes over time, as reviewed by De Boer *et al.*(28) Both classical^30−32^ and steered MD simulations^33,34^ have shown that breathing motions of the AChE gorge allow the natural substrate and other ligands to enter and exit the active site, and that hydrolyzed substrates and other ligands may diffuse from AChE *via* different pathways known as back or side doors.^35−37^ The Ω loop of AChE (Cys69_h_-Cys96_h_ for *h*AChE) has drawn particular interest because it is homologous to the activation loop of the hydrolase protein *Candida rugose* lipase, which belongs to the same α/β hydrolase-fold superfamily as AChE. Structural studies of this hydrolase have shown that its activation loop blocks the active site until a lipid substrate approaches.^38,39^ Site directed-labeling and time-resolved fluorescence anisotropy in combination with MD simulations have also clarified the dynamic behavior of the gorge’s rim,^40^ in particular the Ω loop.^41,42^ This supports the hypothesis that the Ω loop similarly regulates the opening and closing of the gorge to control substrate binding and product ejection in AChE.

Here, we investigate the dynamics of AChE aiming to identify factors determining the potency and selectivity of inhibitors targeting AChE1 in order to evaluate AChE1’s potential as a safe insecticidal target for vector control. To this end, MD simulations of the *Anopheles* AChE1 (*Ag*AChE1) and mouse AChE (*m*AChE) were performed to characterize and compare their structures and dynamics, both as free proteins and as complexes with various noncovalent AChE1 inhibitors having different inhibition and selectivity profiles. The complexes were selected based on experimental data including *in vitro* assay results and X-ray crystallography. Finally, the binding energy contributions of individual amino acid residues and inhibitors were calculated using the MM-PBSA method to generate SARs for potency and selectivity toward AChE1 over *m*AChE.

## Results and Discussion

### Comparison of the Structures and Amino Acid Sequences of *Ag*AChE1 and *m*AChE

The tertiary structures of *Ag*AChE1 and *m*AChE are highly similar, with an amino acid sequence identity of 49% and a sequence similarity of 65%. The active site gorge is structurally conserved and the amino acid residues of the active site, including the catalytic triad, are identical in both species. Differences exist in the three loops at the peripheral site lining the entrance of the gorge (Figure 1 and Tables S1–S3). The sequence identity and similarity of the Ω loop between *Ag*AChE1 and *m*AChE are 60 and 63%, respectively, and a comparison of the corresponding crystal structures^43,44^ indicates that both loops adopt very similar conformations. One notable sequence difference is that Ile231_Ag_ in *Ag*AChE1 is replaced by the bulkier Tyr72_m_ in *m*AChE, so the gorge opening in *m*AChE is narrower than in *Ag*AChE1. Furthermore, there are differences in the presence and positioning of prolines in the Ω loop. The conformations of loops 1 and 2 differ between *Ag*AChE1 and *m*AChE, partly because their lengths differ. Loop 1 in *Ag*AChE1 is three amino acids shorter than in *m*AChE and has 42% sequence identity (with 50% similarity). Alternative loop 1 conformations have been reported in other complexes, for example in crystal structures of paraoxon-inhibited *h*AChE^45^ and AChE from the electric ray *Torpedo californica* (*Tc*AChE) in complex with tacrine-benzofuran inhibitors.^46^ The tryptophan at the beginning of the loop, which is important for ligand binding, is conserved between the species (Trp441_Ag_/Trp286_m_), but the relatively bulky Phe295_m_ of loop 1 in *m*AChE corresponds to the previously mentioned Cys447_Ag_ residue in *Ag*AChE1, whose utility as a target for the development of selective AChE inhibitors has been debated.^16,17^ Loop 2 is one amino acid residue longer in *Ag*AChE1 than in *m*AChE and the residue Tyr494_Ag_ at the entrance of its binding gorge corresponds to the smaller Gly342_m_ in *m*AChE (Table S3). Only one amino acid residue in loop 2 is conserved between *Ag*AChE1 and *m*AChE: a lysine in the middle of the loop near the Ω loop (Lys501_Ag_/Lys348_m_). Despite the low sequence identity of loop 2 (7%), its sequence similarity is high (43%). The α-helix adjacent to loop 2 lines the binding gorge and, as mentioned, the residues directed toward the active site are conserved (Table S3). There are also notable differences between *m*AChE and *Ag*AChE1 in terms of the distribution of proline residues in loops 1 and 2. Overall, however, the ternary structures and the sequences of *Ag*AChE1 and *m*AChE are highly similar.

### Dynamics of *Ag*AChE1 and *m*AChE

The dynamics of *Ag*AChE1 and *m*AChE were studied to explore their conformational landscapes in the absence of inhibitors. Five parallel 500 ns MD simulations were performed for each apo enzyme using different initial velocities, all of which converged within 50 ns as demonstrated by their root-mean-square deviation (rmsd) values (Figure S1). The trajectories of the last 450 ns of each simulation were thus analyzed, giving a total of 2.25 μs of simulated data per apo enzyme. This enabled thorough exploration of the enzymes’ main chain movements in addition to the side-chain dynamics that were examined in previously reported studies of AChE based on shorter MD simulations.^28^

**Figure 2:**
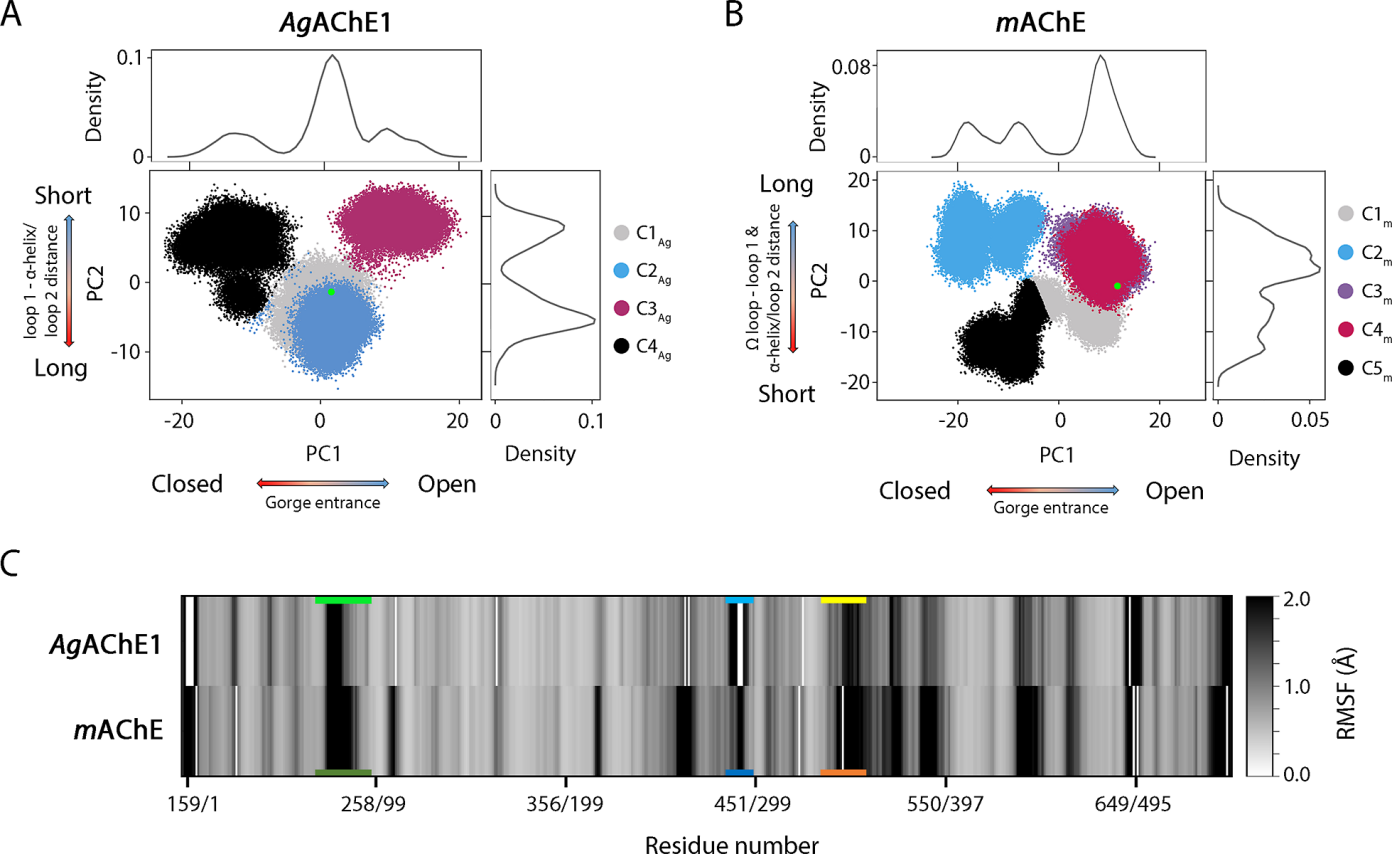
Global and local dynamics of apo AChEs. Projections of the first two PCs of the combined trajectories for *Ag*AChE1 (A) and *m*AChE (B), respectively. The largest collective motion of the enzymes can be seen along PC1, and the second largest along PC2, and the motions were dominated by the three loops lining the gorge entrance of AChE. The colors show the distribution of different clusters in the PCA subspace. Each cluster represent a conformational state of AChE that is highly populated over time. The green dot represents the starting X-ray crystal structure [PDB: 5X61 (*Ag*AChE1) and 1J06 (*m*AChE)]. The histograms visualize the relative number of conformations in the clusters of the PCA subspace. (C) Comparison of fluctuations of amino acid residues based on RMSF values of the protein backbone. RMSF values ≥ 2 Å are colored in black, and gaps in sequence alignment in white. The amino acid residue numbers are reported as *Ag*AChE1/*m*AChE. Residues marked with green, blue, and yellow are present in the Ω loop, loop 1, and α-helix/loop 2, respectively. The analyses were performed on the main chain atoms over the 50–500 ns for the combined simulations.

The largest collective motions of the enzymes were also investigated by performing principal component analysis (PCA) of the main chain atom coordinates of the combined trajectories of the *Ag*AChE1 and *m*AChE simulations. Plots of the resulting principal components (PCs) showed that *m*AChE was more dynamic than *Ag*AChE1 (*i.e.*, the main chain atoms in *m*AChE moved more compared to the atoms in *Ag*AChE1) and accordingly had higher PC eigenvalues (Figures 2A,B and S2). Moreover, the calculated root-mean-square fluctuation (RMSF) values for each residue in the combined trajectories for *m*AChE were larger than those for *Ag*AChE1 (Figure 2C). A previous computational study comparing the dynamics of *Tc*AChE to *m*AChE similarly indicated that *m*AChE was more dynamic, and the authors concluded that this difference affected the accessibility of the active site to covalent inhibitors.^47^ However, this earlier analysis was mainly based on short MD simulations (20 ns), which complicates comparisons to the present work.

**Figure 3:**
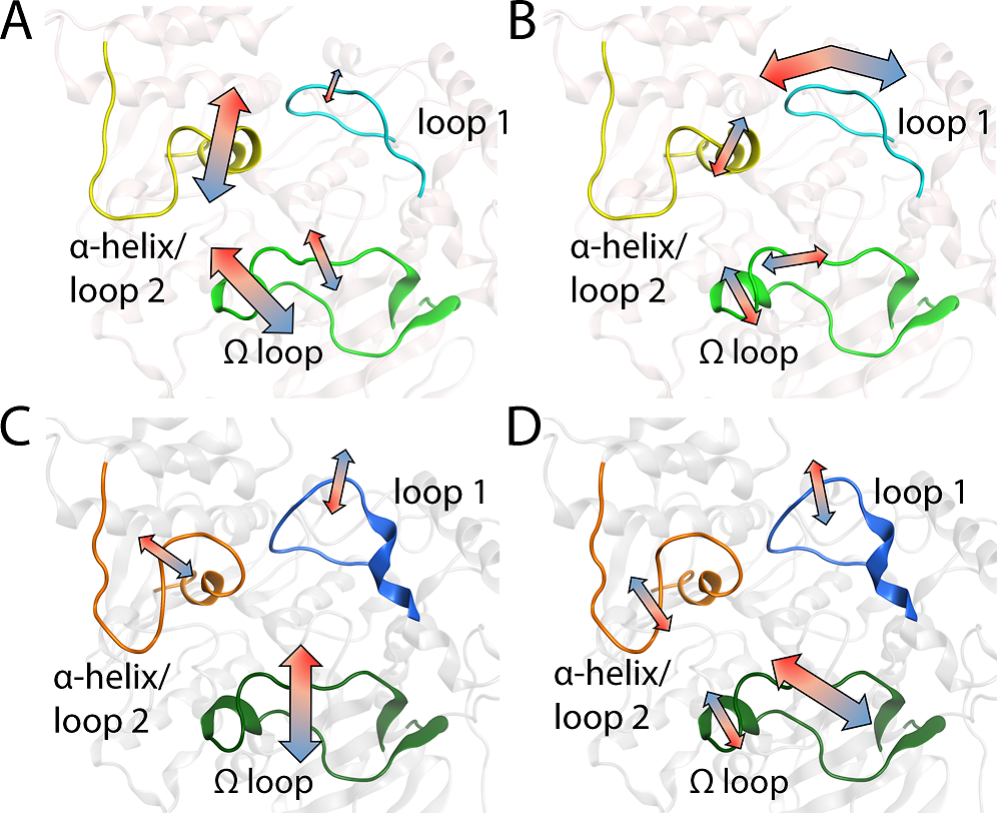
Illustration of the major collective motions along the PC1 and PC2 of *Ag*AChE1 (A,B) and *m*AChE (C,D). The directions of the motions are shown by arrows marked by a color gradient red to blue representing negative to positive values PC values shown in Figure 2. The magnitude of motions are depicted by the length and thickness of the arrows (long and thick arrows represent large motions and vice versa). (A) The gorge of *Ag*AChE1 was opened and closed by movements of the Ω loop and loop 2 with respect to loop 1. (B) The twisting motion of loop 1 around the gorge entrance differentiates the open and closed conformations from the intermediate states. (C) The gorge of *m*AChE was opened and closed by movements of the Ω loop and loop 1. The movements of the Ω loop differentiate the two closed states from each other (D). The figures are based on protein coordinates of previously published crystal structures (PDB codes 5X61 and 1J06, for *Ag*AChE1 and *m*AChE, respectively).

The largest collective motion (represented by the first PC of the PCA, *i.e.*, PC1) for both enzymes corresponded to an opening and closing of the gorge entrance. Whereas previously described breathing motions mainly involved side-chain movements,^28^ this movement involved much more pronounced main chain motions that made the catalytic triad accessible at the bottom of the gorge. Distance calculations and visualizations of these extreme motions revealed that the movement predominantly involved the Ω loop, loop 1, and loop 2 (Figures 3 and S3). The movements of loop 2 also affected the adjacent α-helix, so these two structural elements are jointly referred to as α-helix/loop 2 and their movements are analyzed collectively henceforth. The Ω loop exhibited particularly pronounced dynamics, in accordance with earlier suggestions based on both experiments and calculations.^41,42^ These substantial movements may appear inconsistent with the surprisingly well-defined electron density of the Ω loop seen in X-ray crystal structures, but the apparent discrepancy can be attributed to loop-stabilization induced by crystal packing.^48^ The clear differences between the dynamics of *Ag*AChE1 and *m*AChE are largely attributable to the interplay between these three loops and how it affected the collective motions of the two enzymes (Figure 3).

**Figure 4:**
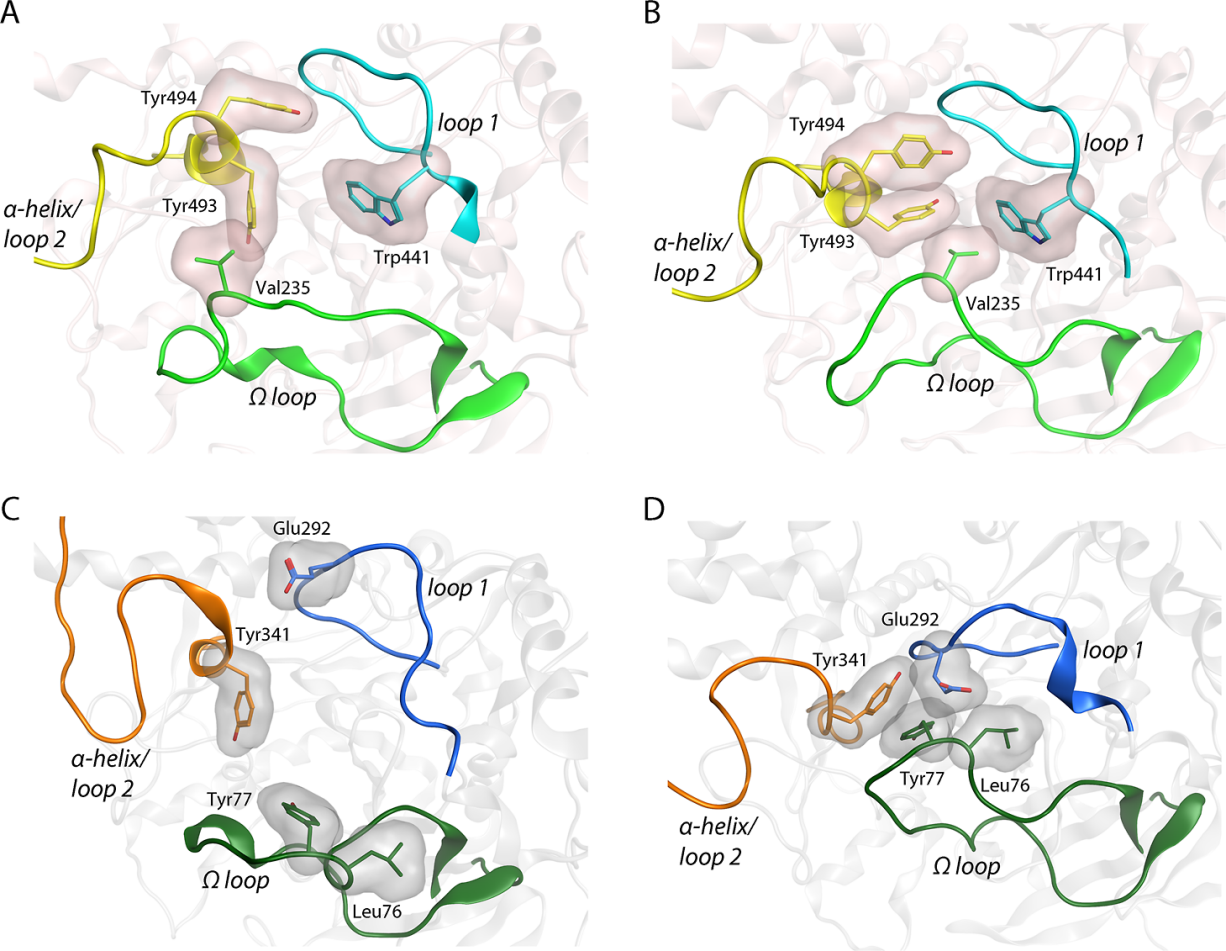
Examples of open and closed conformations adopted by *Ag*AChE1 and *m*AChE during the MD simulations, based on the clustering of largest collective motions. *Ag*AChE1 in the open (A) and closed (B) conformations found in clusters C3_Ag_ and C4_Ag_, respectively. *m*AChE in the open (C) and closed (D) conformations found in C4_m_ and C2_m_, respectively. Ribbons that are part of the three loops are marked with green, blue, and yellow lines for the Ω loop, loop 1, and α-helix/loop 2, respectively. *Ag*AChE1 has lighter ribbon colors than *m*AChE. Key amino acid residues contributing to the closing of the gorge are displayed with carbons colored according to the loops, oxygen in red, and nitrogen in blue.

To identify ensembles of local conformational states of *Ag*AChE1 and *m*AChE within their conformational landscapes, clustering was performed based on the first three PCs (Figure 2A,B and Tables S4, S5). Clustering of *Ag*AChE1 yielded four clusters. The most populated of these clusters were C1_Ag_ and C2_Ag_, which accounted for 40 and 20% of the extracted conformations, respectively, and corresponded to intermediate conformational states that were neither fully opened nor fully closed. Notably, the projection of the X-ray crystal structure of *Ag*AChE1 (PDB 5X61) falls into the most populated space of the conformational landscape (Figure 2A). The intermediate states differed from the open and closed states (C3_Ag_ and C4_Ag_) in terms of the positioning of the Ω loop and the α-helix/loop 2 but also exhibited a displacement of loops 1 and 2 that caused these loops (including their subdomains S3 and S2) to be rotated at the gorge entrance (Figures 3 and S4, S5). The two intermediate states differed in terms of the position of the Ω loop in relation to loop 1. The gorge entrance was most widely opened (C3_Ag_; 20%) when the Ω loop moved away from the binding gorge and the distance between α-helix/loop 2 and loop 1 was greatest, while the closed state (C4_Ag_; 20%) occurred when both the Ω loop and loop 2 were in contact with loop 1 (Figures 3 and S4, S5). Analysis of side chains of the residues in the three loops revealed that Val235_Ag_ (Ω loop), Trp441_Ag_ (loop 1), Tyr493_Ag_, and Tyr494_Ag_ (α-helix/loop 2) in *Ag*AChE1 contributed to the closing of the gorge by blocking the entrance (Figure 4A,B).

In contrast to the conformational state distribution of *Ag*AChE1, *m*AChE had three open (C1_m_, C3_m_, and C4_m_; 23, 19, and 19%) and two closed states (C2_m_ and C5_m_; 20 and 19%) whose positions along PC1 are shown in Figure 2B. Additionally, the projection of the X-ray crystal structure of *m*AChE (PDB 1J06) falls into the most populated space of its conformational landscape, *i.e.* the open state. The fact that the crystal structure conformations of both AChE’s exist within highly populated conformational space contradicts claims in the field that crystallization conditions favor a narrow gorge conformational state.^41,42^ Instead, it appears that the crystal structures adopt low energy conformational states resembling those favored in simulations. The opening and closing of the *mAChE* gorge entrance were mainly due to movements of the Ω loop and loop 1 at S2, although loop 2 at S3 was also dynamic (Figures 3 and S6, S7). In the open states, the Ω loop was separated from both loop 1 and loop 2, making the gorge accessible. In the closed states, the gorge entrance was narrowed by successive close contacts between the Ω loop and loop 1 due to movements of the former. Substates of the closed and open conformational states were distinguished by the positioning of the Ω loop. C1_m_ differs from the other two open states (C3_m_ and C4_m_) due to a displacement of the Ω loop with respect to loop 1, resulting in a slightly narrower gorge opening than in C3_m_ and C4_m_ (PC2). The conformational state C4_m_ exhibits a wide gorge opening due to a displacement of the Ω loop relative to loop 2 and rearrangement of the Ω loop (Figure 4C). In both closed states, the Ω loop and loop 2 were closer together for C2_m_ compared to C5_m_, where the side chains of Leu76 and Tyr77 in the Ω loop, Glu292 in loop 1 and Tyr341 in the α-helix/loop 2 blocked the entrance of the gorge (Figure 4D).

The PCA and subsequent clustering revealed that opening and closing motions occur at the gorge entrance in both *Ag*AChE1 and *m*AChE. However, the gorge entrance was significantly wider for *m*AChE, as can be seen when comparing distances between the Ω loop and loops 1 or 2 for *Ag*AChE1 and *m*AChE (Figure S3). Moreover, the opening and closing motions occur *via* different mechanisms in each protein due to the differing dynamics of the three loops (Figure 3). We therefore attempted to relate the differences between the largest collective motions of *Ag*AChE1 and *m*AChE to differences in the amino acid sequence of their loops. This suggested that differences in proline position and content may be responsible for the mechanistic differences of the motions. In *Ag*AChE1, twisting motions dominated the most dynamic part of the Ω loop because Pro240_Ag_ is next to the α-helix segment, restricting movements with larger amplitudes. In *m*AChE, the corresponding Pro78_m_ is shifted down three amino acids in the loop sequence, allowing a more pronounced opening and closing motion of the full Ω loop with a greater amplitude. Pro450_Ag_ of *Ag*AChE1 is positioned at the very end of loop 1, which allows the loop to move with a twisting motion despite being shorter than that in *m*AChE. In *m*AChE the corresponding Pro290_m_ is instead located at a central position of loop 1. This, together with the loop being three residues longer than in *Ag*AChE1, allows loop 1 in *m*AChE to move toward (and away from) the Ω loop. The positioning of Pro290_m_ thus restricts the twisting motion but enables a larger motion of the loop in *m*AChE. Loop 2 in *Ag*AChE1 also undergoes a pronounced twisting motion in which the loop’s conformation changes during the largest collective motions. This loop does not undergo such twisting motions during the largest collective motions of *m*AChE; instead, its motions largely correlated with those of the S3 subdomain. The amino acid sequence of loop 2 in *m*AChE is one residue shorter than in *Ag*AChE1 but contains a proline residue (Pro344_m_) that is absent in *Ag*AChE1, explaining the substantial differences in the dynamics of the two loops.

### Dynamics of Active Site Gorge

**Figure 5:**
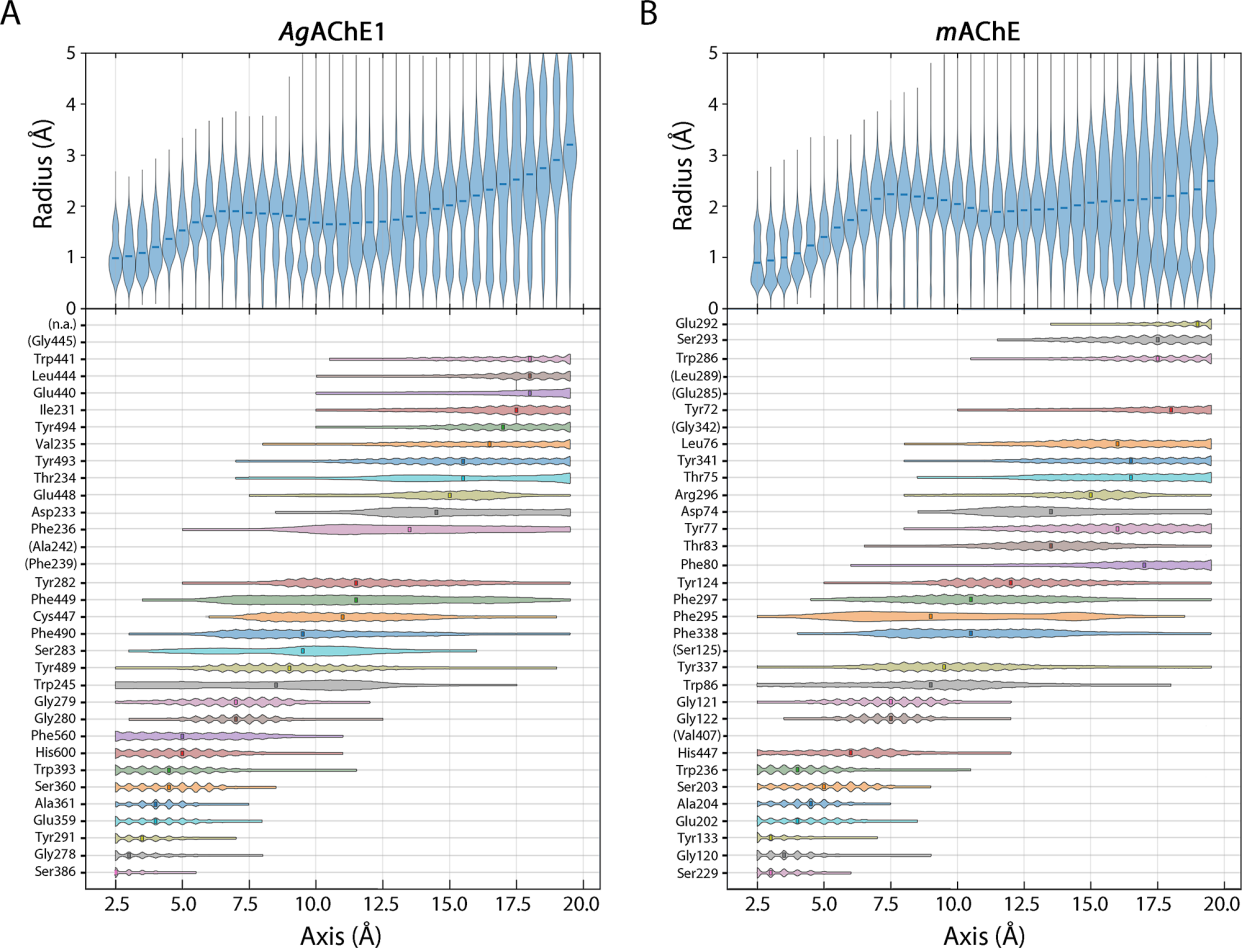
Characterization of the gorge shapes of the combined trajectories of the *Ag*AChE1 and *m*AChE simulations, respectively. Gorge radius distribution along the gorge axis (2.5–20 Å) including the position distribution of amino acid residues present at the surface of the gorge are shown for *Ag*AChE1 (A) and *m*AChE (B), respectively.

The PCA and subsequent clustering showed that *m*AChE exhibits more pronounced conformational dynamics than *Ag*AChE1 but did not explain how the observed differences affected the active site gorge. Therefore, to characterize the shapes of the gorges, we calculated the radius of the gorge over the combined trajectories of the *Ag*AChE1 and *m*AChE MD simulations (Figures 5 and S8). In this context, the radius is the distance across the principal axis (*i.e.*, the axis extending from the bottom of the gorge to the opening) between amino acid residues lining the gorge’s periphery. The radius fluctuated between 0.5 and 5 Å, revealing that the gorge shape was dynamic in both *Ag*AChE1 and *m*AChE. Both enzymes had similar gorge radii and fluctuations between the bottom of the gorge and the catalytic site, *i.e.*, between 0 and 7.5 Å along the principal axis. Within this region, the radius increased steadily from approximately 0.5 Å at the bottom to 2.8 Å at the catalytic site. The amino acid residues lining the gorge of this region were also similar in the two enzymes and included the catalytic residues Ser360_Ag_/Ser203_m_ and His600_Ag_/His447_m_. The dynamics of the middle section of the gorge (from 7.5 to 13.5 Å along the principal axis) differed substantially between the two enzymes, with *Ag*AChE1 showing more pronounced fluctuations in radius than *m*AChE. This region extended from Gly279_Ag_/Gly121_m_*via* Tyr489_Ag_/Tyr337_m_ and Trp245_Ag_/Trp86_m_ to Tyr282_Ag_/Tyr124_m_. Interestingly, the gorge-lining residues in this region include Cys447_Ag_/Phe295_m_ as well as the amino acid residues of the dynamic Ω loop, which may account for the observed differences. In accordance with this hypothesis, Carlier *et al.* proposed that the high selectivity of β- and γ-branched 1-alkylpyrazol-4-yl for inhibition of *Ag*AChE1 *vs h*AChE was due to the more pronounced fluctuations of Trp245_Ag_ compared to the corresponding Trp86_h_.^14^

In the upper region, which extends from 13.5 Å along the principal axis to the top of the gorge at 20 Å, *m*AChE underwent substantially greater fluctuations than *Ag*AChE1. In this region, the gorge entrance is lined by amino acid residues from all three loops including Trp441_Ag_/Trp286_m_, Tyr494_Ag_/Gly342_m_ and Ile231_Ag_/Tyr72_m_ from loop 1, α-helix/loop 2, and the Ω loop, respectively.

The gorge characterization also revealed that the differences between the amino acid sequences of the two species affected the shape of the binding pocket (Figure 5). Analysis of the 3D structures of the conformers indicated that the main effect relates to the internal placement of the α-helix lining the gorge, which differs structurally between *Ag*AChE1 and *m*AChE. In the *Ag*AChE1 simulations, the Tyr494_Ag_ at the beginning of loop 2 (within subdomain S3 at the top of the gorge) formed a close interaction with Ile446_Ag_ in loop 1 at subdomain S2 at the peripheral site (Figures S9 and S10A,B). This interaction is not present in *m*AChE because Tyr494_Ag_ is replaced by Gly342_m_. Distance calculations between the two residues over the trajectories confirmed that the distances between loop 1 and loop 2 in *m*AChE were longer and varied more over time than those in *Ag*AChE1 (Figure S10C,D). In the lower part of the gorge of *Ag*AChE1, the gorge-lining α-helix was anchored *via* two hydrogen bonds that link the main chain atoms of Gly487_Ag_ and Tyr488_Ag_ to Asp552_Ag_ in the adjacent α-helix within S3 closer to the surface (Figures S11 and S12). These hydrogen bonds cannot form in *m*AChE because Asp552_Ag_ is replaced by Ser399_m_. Taken together, these results indicate that sequence differences between the two enzymes alter the amino acid residue interactions within the proteins, which in turn affects the internal positioning of the α-helixes and their dynamics in the active site gorge.

### Inhibitors of *Ag*AChE1 and *m*AChE

**Figure 6:**
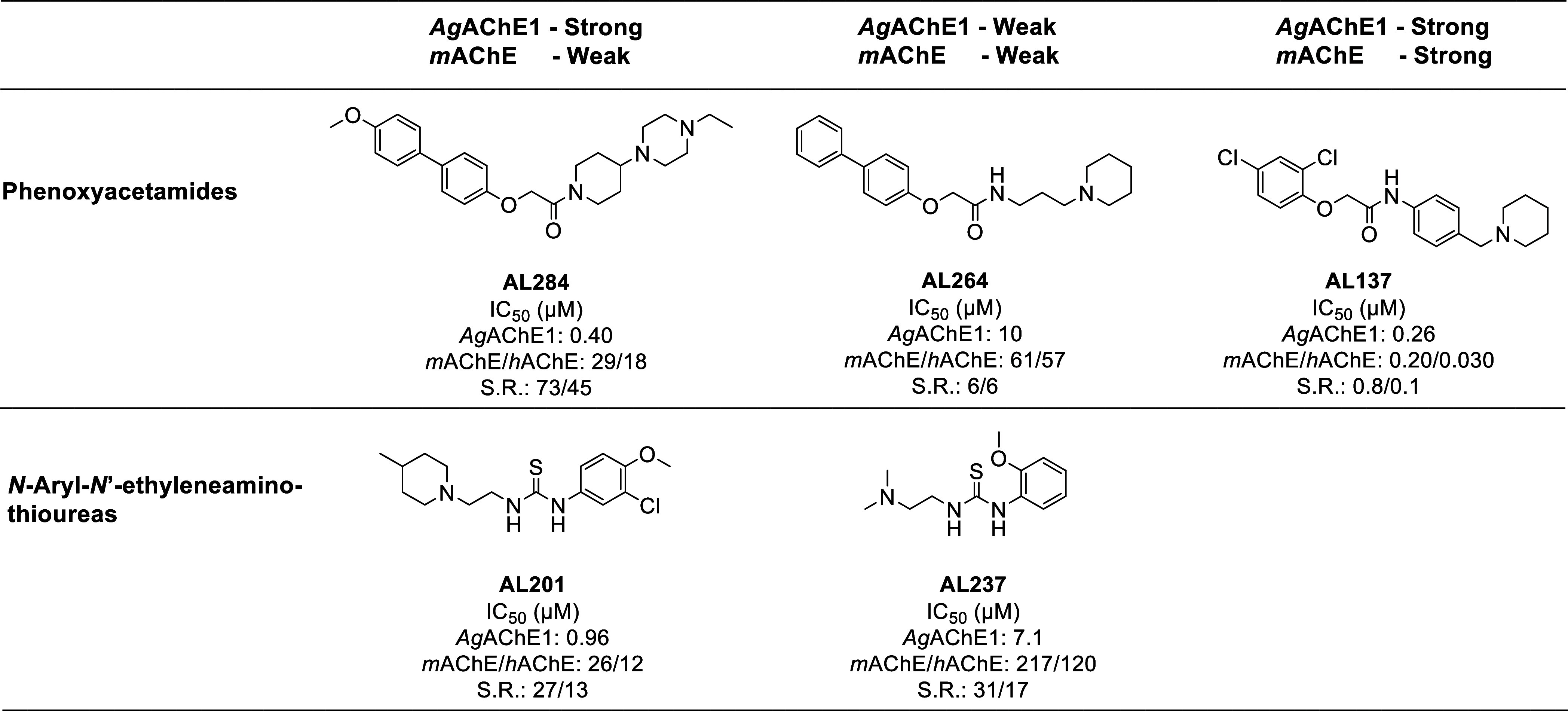
Chemical structures, IC_50_ values and selectivity ratios (S.R.) of investigated inhibitors.

Ten *Ag*AChE1•inhibitor and *m*AChE•inhibitor complexes were studied to characterize their dynamics and binding interactions, and subsequent relationships to the inhibitors’ potency and selectivity profiles. The set comprised of five inhibitors belonging to two compound classes: phenoxyacetamide-based inhibitors^20,22^ and *N*-Aryl-*N*′-ethyleneaminothioureas^21^ (Figure 6). All of them have extended rod-like molecular shapes with aromatic fragments at one end and aliphatic amine fragments at the other, which are assumed to be protonated at physiological pH. The compounds differ in terms of molecular size and number of rotatable bonds. The set included strong and weak inhibitors of *Ag*AChE1 from both classes (IC_50_ < 1 μM and IC_50_ > 5 μM, respectively, based on experimental inhibition measurements) for which X-ray crystallographic data on the *m*AChE•inhibitor complex was available, providing experimentally based starting conformations for the MD simulations. The IC_50_ values had been determined by the same laboratory and at comparable conditions,^20−22^ justifying the comparison of potency. Furthermore, determination of *K*_*i*_ values for other inhibitors with similar overall chemical structure (rod-like with aromatic- and aliphatic amine fragments) showed good agreement with the IC_50_ values using these conditions,^24,49,50^ which further supports the relevance of the method. In total the set included three strong and two weak inhibitors of *Ag*AChE1, with one compound being a strong inhibitor of *m*AChE and four being weak inhibitors of this enzyme.

**Figure 7:**
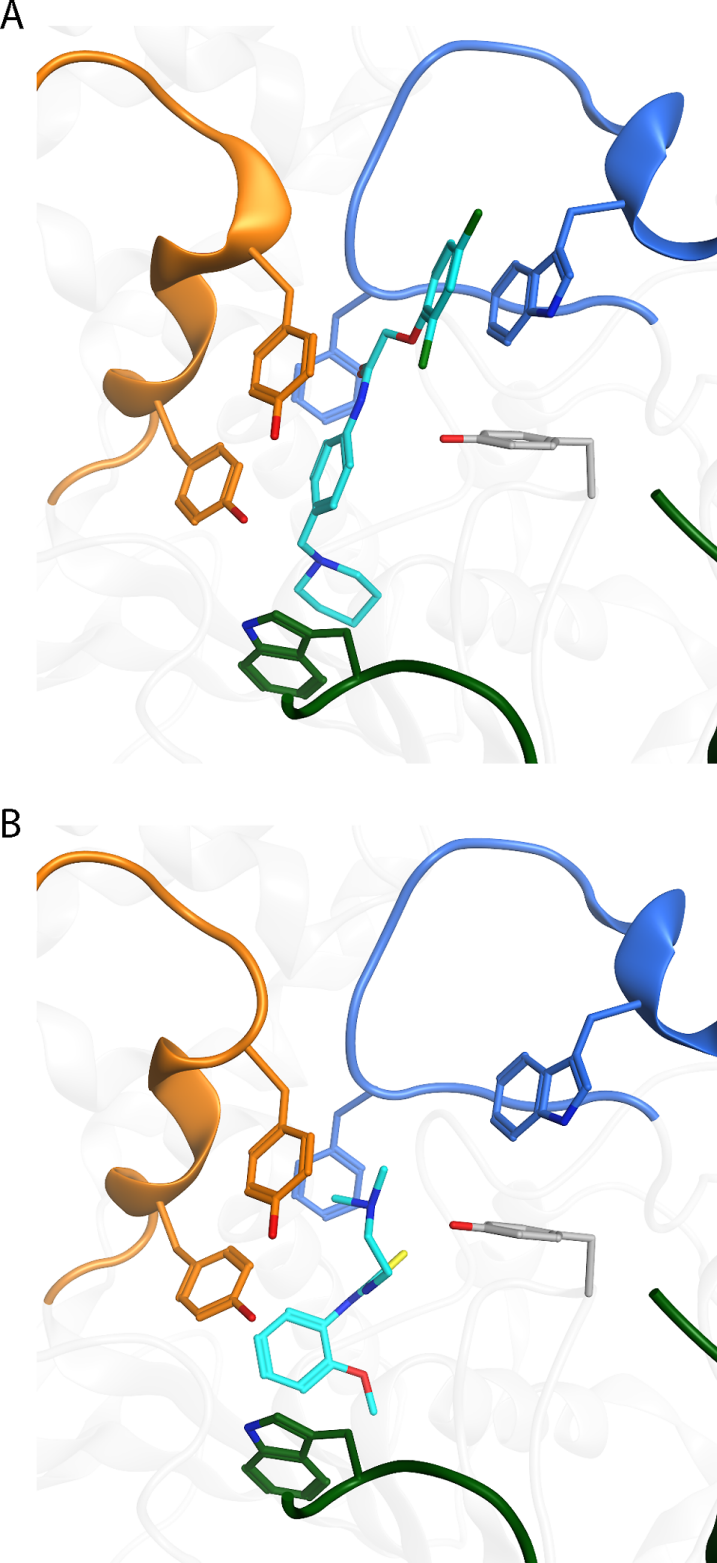
Binding poses of inhibitors AL137 (A) and AL237 (B) in complex with *m*AChE determined by X-ray crystallography. *m*AChE is displayed with gray ribbons with the Ω loop, loop 1, and α-helix/loop 2 colored in dark green, blue, and orange, respectively. Amino acid residue carbon atoms are colored according to the ribbons, and inhibitors’ carbon atoms are colored in cyan. Oxygen-, nitrogen-, sulfur- and chlorine atoms are colored in red, blue, yellow, and green, respectively. The crystal structure of complex *m*AChE•AL137 has been previously published (PDB code 5FOQ), while *m*AChE•AL237 is presented here (PDB code 8ORC).

X-ray crystal structures were available for all inhibitors in complex with *m*AChE, except for AL237 and AL201. The crystal structure of *m*AChE•AL237 was determined during this work (Table S6 and Figure S13). Unfortunately, disordered electron densities were present in multiple collected X-ray data sets for *m*AChE•AL201, so no X-ray crystal structure could be determined for this complex. This inhibitor was therefore modeled *in silico* in the binding site of *m*AChE, taking the experimentally determined electron density map into account. All inhibitors bind to the active site gorge according to the electron density maps, and thus show a similar mode of action for inhibition. All of the *m*AChE residues whose side chains interacted with the inhibitors are conserved in *Ag*AChE1 in terms of both sequence and location within the apo 3D structure. The modeled *Ag*AChE1•inhibitor complexes thus display the same interaction patterns as are seen in the X-ray crystal structures of *m*AChE. Analysis of the structures of the *m*AChE•inhibitor complexes revealed that all inhibitors except AL237 bind in an extended conformation spanning the gorge (Figures 7 and S14).

### Inhibitors’ Influence on the Dynamics of *Ag*AChE1 and *m*AChE

Three MD simulations of 200 ns were performed for each of the five inhibitors in complex with *Ag*AChE1 and *m*AChE. The simulations all converged within 50 ns (based on the rmsd values of *Ag*AChE1 and *m*AChE main chain atoms, Figures S15 and S16), so the final 150 ns were used for further analysis. In contrast, the rmsd values of the inhibitors’ heavy atoms fluctuated both within and between simulations, indicating that the inhibitors adopted multiple different binding modes in the gorges of the enzymes (Figures S17 and S18).

**Figure 8:**
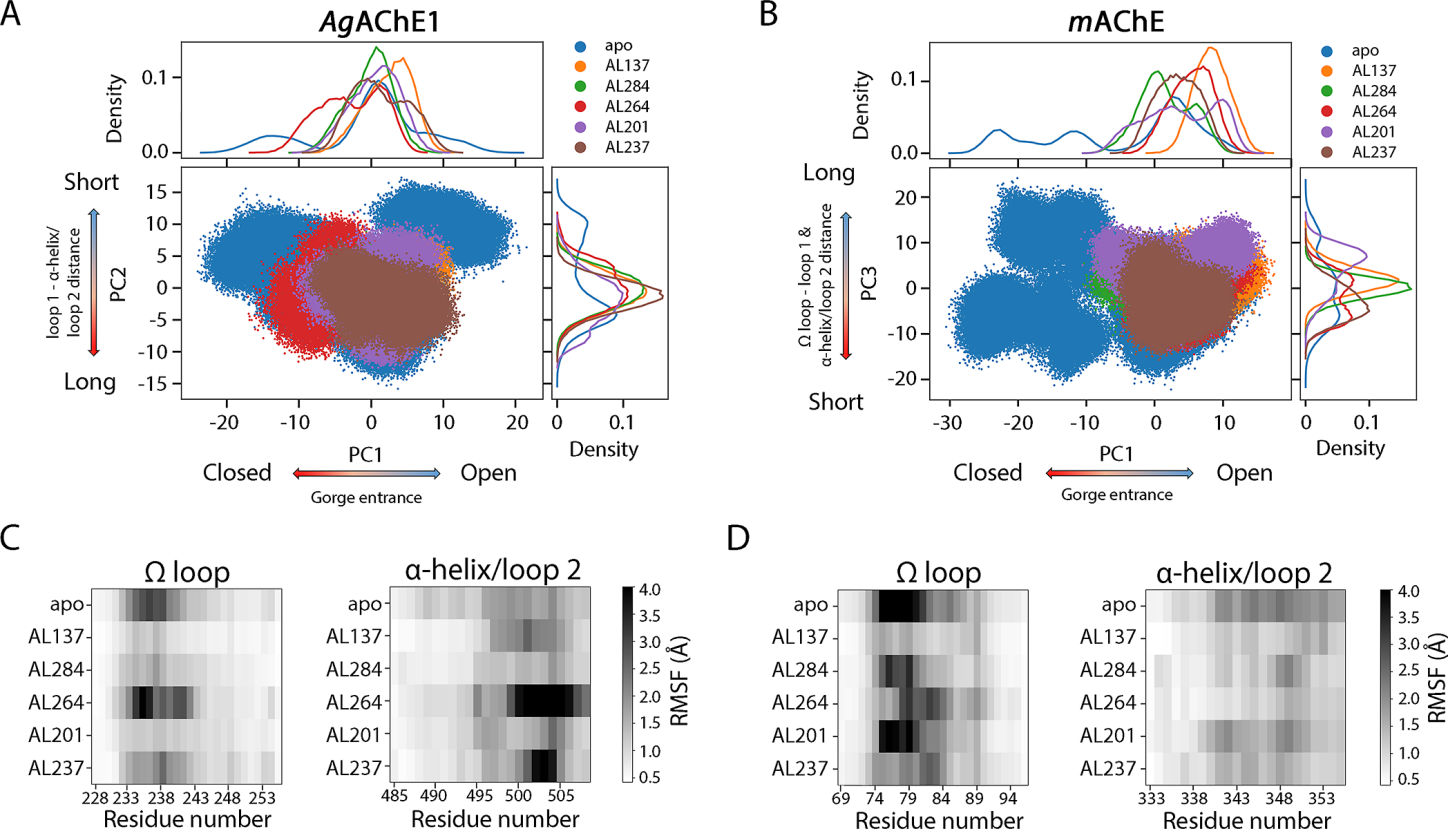
Global and local dynamics of AChE•inhibitor complexes. Projections of two PCs of the combined trajectories of apo *Ag*AChE1 and the *Ag*AChE1•inhibitor complexes (A), and apo *m*AChE and the *m*AChE•inhibitor complexes (B). The largest collective motion of the enzymes can be seen along PC1 and the second largest along PC2. The motions of the displayed PCs were dominated by the three loops lining the gorge entrances of AChEs. The colors show the distribution of different complexes, and the histograms visualize the relative amount of conformations of AChEs in the PCA subspaces. Comparison of fluctuations of amino acid residues in the Ω loop, loop 1, and α-helix/loop 2 for *Ag*AChE1 (C) and *m*AChE (D), respectively, including the apo and inhibited enzymes. RMSF values ≥ 4 Å are colored in black. The analyses were performed on the main chain atoms over the 50–500 ns or 50–200 ns for the combined simulations of *Ag*AChE1 and *m*AChE, respectively.

To clarify the impact of bound inhibitors on the conformational dynamics of *Ag*AChE1 and *m*AChE, we performed PCAs on the main chain atom coordinates from the combined trajectories of inhibitor-bound and apo *Ag*AChE1 and *m*AChE (Figure 8A,B). As in the apo enzymes, the opening and closing of the gorge entrance were the largest collective motions for both sets of complexes: the correlation coefficient between the first principal component (PC1) of the complexes and the apo enzyme was 0.94 for *Ag*AChE1 and 0.92 for *m*AChE. However, the magnitude of the dynamics of the apo enzymes was substantially greater than that of the inhibitor complexes, indicating that inhibitor binding considerably restricted the opening and closing motion (Figure 8A,B). The inhibitor-bound enzymes populated the dominant conformational states of the corresponding apo structures, *i.e.*, the intermediate states for *Ag*AChE1 and the open states for *m*AChE (Figure 8A,B), which is also where the X-ray crystal structures were projected. The patterns of the collective motions along PC2 and PC3 differed between the complexes of the two enzymes. As in the apo enzyme, the second largest collective motion of inhibitor-bound *Ag*AChE1 was dominated by the distance between loop 1 and α-helix/loop 2; the correlation coefficient between the inhibitor complexes and the apo enzyme was 0.90. However, the third largest collective motion differed between the apo enzyme and the inhibitor complexes: in the complexes, the Ω loop moved with respect to both loop 1 and α-helix/loop 2. This movement was most pronounced for the *Ag*AChE1•AL264 complex, which had the longest distances between these structural elements (Figure S19). The second largest collective motion of the *m*AChE complexes involved movement of the modeled Pro258_m_-Gly264_m_ loop and is considered less relevant to the research question. This movement was much less profound in apo *m*AChE. The corresponding loop in *Ag*AChE1 is shorter than in *m*AChE, differs in sequence, and did not impact the collective motions. The third largest collective motion of the *m*AChE-inhibitor complexes resembled the second largest collective motion of apo *m*AChE but the enzyme complexes were less dynamic. The largest motions identified in the PCAs were analyzed closely to identify differences in the AChE dynamics induced by potent or weak inhibitors. However, no discriminative pattern could be identified on this basis.

To better understand the inhibitors’ effects on local enzyme dynamics, the fluctuations of the residues of the three loops were studied. The RMSF values of *Ag*AChE1 clearly showed that the dynamics of the Ω loop and loop 2 were strongly affected, while loop 1 appeared to be less affected by the inhibitors (Figures 8C and S20). The residues of the Ω loop were less dynamic when strong inhibitors (AL137, AL284, and AL201) were bound. The weakest inhibitor, AL237, also induced some rigidity in the Ω loop, while AL264 instead induced increased this loop’s fluctuations; these findings are consistent with the PCA analysis. Loop 2 of *Ag*AChE1 exhibited substantially more pronounced dynamics when the weak inhibitors (AL264 and AL237) were bound but the strong inhibitors (AL137, AL284, and AL201) did not greatly alter its dynamics.

The inhibitors also affected the fluctuations of the residues of the Ω loop of *m*AChE (Figures 8D and S21B). The strongest effect was seen for the strong inhibitor AL137, which reduced the loop’s fluctuations substantially. AL237 and AL264 also altered the loop’s dynamic pattern by enhancing the fluctuations in some parts and weakening them in others, but AL284 and AL201 had less impact on the Ω loop. In *m*AChE, the inhibitors did not alter the dynamics of loop 1 and loop 2 to any great extent (Figures 8D and S21C,D).

In summary, binding of inhibitors in the active site gorge restricts the dynamics of AChE relative to what is seen in the apo enzymes. The formation of a soman-Ser203 adduct was previously reported to induce thermodynamic and conformational stabilization of *h*AChE, particularly in the region of the active site.^37^ A closer analysis of our strongest *Ag*AChE1 inhibitors showed that they stabilized the Ω loop and did not induce fluctuations of loop 2, unlike the two weak inhibitors. The only potent inhibitor of *m*AChE, AL137, also stabilized the Ω loop, which may indicate that weakening the dynamics of this loop is a general characteristic of strong inhibitors. The effects of weak inhibitors on the enzyme dynamics appeared to differ between the two species. In *Ag*AChE1, weak inhibitors amplified the fluctuations of loop 2, which was not seen in *m*AChE. As previously mentioned, the dynamics of loop 2 in *m*AChE are less pronounced than in *Ag*AChE1 because it is shorter and has Pro344_m_ positioned close to the adjacent α-helix, which is absent in *Ag*AChE1.

### Water Molecules in the Gorges of *Ag*AChE1 and *m*AChE

The active site gorges of the apo enzymes are populated with water molecules (Figures S22 and S23). It has been shown that waters move in and out through the main entrance once every 200 ps, on average,^51^ and the importance of conserved waters for ligand binding has been highlighted.^52^ In order to investigate the water population in the gorges of *Ag*AChE1 and *m*AChE, we examined conserved waters in 40 crystal structures of apo AChEs and AChE•inhibitor complexes (mouse and human enzymes, Tables S7 and S8). The putative hydrogen boding pattern between waters and amino acid residue atoms in the active site gorge were studied and 13 putative hydrogen bonds were selected for water analysis of the trajectories (Table S9 and Figure S24). These 13 water sites were conserved in more than 65% of the investigated complexes, The water molecules’ occupancy at hydrogen bonding distances (<3.5 Å) to 12 amino acid residue atoms of apo *Ag*AChE1 were on average more than 0.65, and one was below 0.35 over the full trajectory (Table S10). Lowering the distance cut off to less than 3 Å, thus indicating stronger hydrogen bonds, led to five sites with more than 0.80 water molecules on average, while the remaining eight sites had below 0.60 water molecules on average (Table S12). The five residue atoms with highest water occupancy at hydrogen bonding distances were the hydroxyl oxygens of Ser283_Ag_ and Tyr282_Ag_ at the lower part and waist of the gorge, respectively, the carbonyl oxygens of Trp242_Ag_ and Ile231_Ag_ in the Ω loop, and the carbonyl oxygen of Glu448_Ag_ in loop 1. Tyr282_Ag_ that is located at the narrow waist of the gorge had the highest occupancy of 2.75 and 1.42 water molecules on average at distances lower than 3.5 and 3 Å, respectively. This site (the narrow waist) has been reported to be the node for transporting waters in and out of the active site of *m*AChE,^51^ thus explaining its high occupancy over time connecting water molecules in the lower active site with water molecules in peripheral site at the entrance of the gorge during exchange of waters. The overall water occupancy in apo *m*AChE defined by the selected residue atoms was similar to *Ag*AChE1, including the high water occupancy defined by Tyr282_Ag_/Tyr124_m_, with average water molecules of 2.67 and 1.41 at distances lower than 3.5 and 3 Å, respectively (Tables S10–S13). Nine amino acid residue atoms of apo *m*AChE had on average more than 0.65 water molecules at a shorter distance than 3.5 Å, while the remaining four residue atoms had 0.50–0.55 water occupancy. Even though the two enzymes showed an overall similarity, the water pattern differed for some of the selected residue atoms. For example, in the lower part of the gorge, *m*AChE had on average 1.01 water molecules closer than 3 Å to the carbonyl oxygen of Gly120_m_, while *Ag*AChE1 had only 0.49 corresponding water molecules. On the other hand, *m*AChE had a lower water occupancy defined by the carbonyl oxygen of Trp86_m_ in the Ω loop compared to *Ag*AChE1 (0.69 *vs* 1.04 at a distance cut off <3 Å). The observed differences in water pattern based on average values for some selected residue atoms for *Ag*AChE1 and *m*AChE aligned well with the observed differences in the shape of the binding pockets during the MD simulations.

Water analysis of the AChE•inhibitor complexes revealed that the largest effect upon ligand binding was a profound decrease in the number of water molecules at hydrogen bond distances to Tyr282_Ag_/Tyr124_m_ (Tables S10–S13). The narrow waist of the gorge has a limited volume to host both inhibitors and water molecules. For the phenoxyacetamide-based inhibitors, the decrease in water occupancy defined by Tyr282_Ag_/Tyr124_m_ was approximately 30% compared to apo enzymes for the strong inhibitors, and 50–60% for the weak inhibitors (distance <3.5 Å). For the thiourea-based inhibitors, the decrease in water occupancy for *Ag*AChE1•AL201 was 31% compared to 53% for the mouse enzyme. The smaller AL237 in complex with *Ag*AChE1 and *m*AChE decreased the number of water molecules at hydrogen bonding distance to Tyr282_Ag_/Tyr124_m_ with 41 and 34%, respectively (distance <3.5 Å). In addition, the water occupancy at hydrogen bonding distances to the carbonyls of Thr242_Ag_/Thr83_m_ and Trp245_Ag_/Trp86_m_ in the Ω loop increased in most AChE•inhibitor complexes compared to the apo enzymes. Detailed analysis showed inhibitor-dependent alterations of the water pattern compared to apo (Tables S10–S13). Hence, no additional general trends or pattern for strong or weak inhibitors were observed.

In summary, the water analysis showed in general a high water occupancy at the selected hydrogen bond distances also during enzyme dynamics, albeit with some differences depending on species and bound inhibitor. A perturbing effect on the conserved water pattern was observed when AChE•inhibitor complexes were compared to apo enzymes, in particular for waters at distances close to Tyr282_Ag_/Tyr124_m_ at the narrow waist of the gorge. The decrease in water molecules close to Tyr282_Ag_/Tyr124_m_ appears to be accompanied by an increased water occupancy at the selected atoms in the Ω loop.

### Interactions between Inhibitors and Enzymes

**Figure 9:**
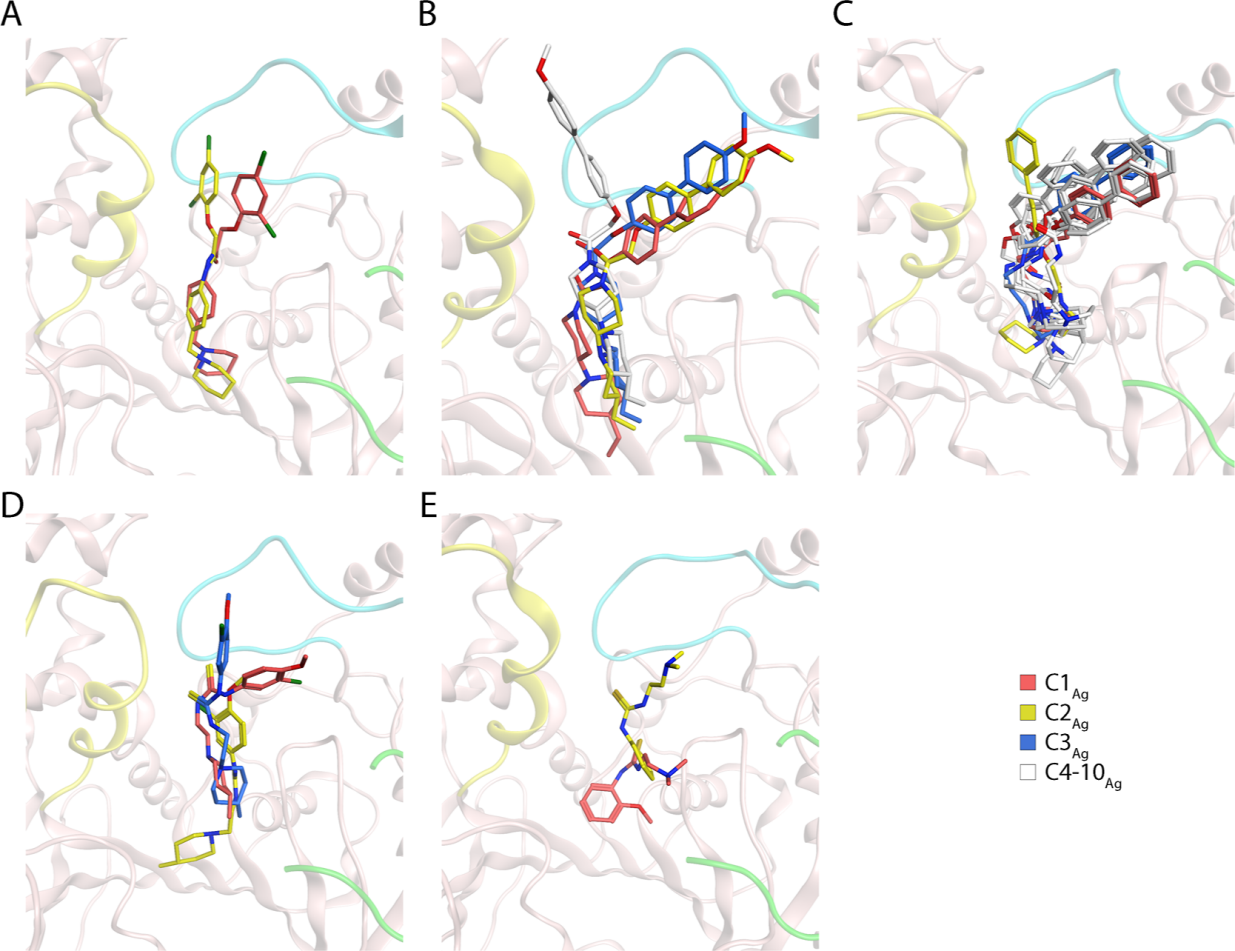
Superposition of conformers of *Ag*AChE1•inhibitors obtained from the cluster analysis performed on protein–ligand interactions using gmx_clusterByFeatures. The inhibitors’ binding poses of the central conformers of each cluster are shown: the phenoxyacetamide-based inhibitors AL137, AL284 and AL264 (A-C), and the *N*-aryl-*N*′-ethyleneaminothioureas AL201 and AL237 (D,E). The protein ribbons shown in the background are the central conformers of the largest clusters, with the Ω loop in light green, loop 1 in cyan, and α-helix/loop 2 in yellow, respectively. Parts of the Ω loop is removed to better display the inhibitor. Oxygen-, nitrogen-, sulfur- and chlorine atoms are colored in red, blue, yellow, and green, respectively.

To further explore the molecular interactions between the inhibitors and enzymes, we developed and implemented a new and fast method to cluster conformers based on their enzyme–inhibitor interaction patterns. This was achieved by measuring distances between amino acid residues of the enzyme and structural fragments of the inhibitor over time (for details, see the Material and Methods section). Our new method is faster than the popular rmsd matrix method because it requires no time-consuming rmsd matrix calculations or superposition to a reference structure. The cluster analysis based on PCA of the reciprocals of the minimum distances revealed fewer enzyme–inhibitor interaction clusters for *Ag*AChE1 than for *m*AChE for all inhibitors (except AL264, which had equal numbers of clusters for both enzymes as shown in Table S14 and Figures 9 and S25). There was no correlation between the number of interaction clusters for an inhibitor and its inhibitory strength.

For the clusters of *Ag*AChE1 in complex with the phenoxyacetamide-based inhibitors, there was a clear difference between the two potent inhibitors AL137 and AL284, and the weak inhibitor AL264, even though all three inhibitors spanned the full extent of the gorge (Figure 9A–C). Two conformational interaction clusters were obtained for AL137 and four for AL284 (with three dominant ones) whereas ten were obtained for AL264. The *N*-Aryl-*N*′-ethyleneaminothioureas AL201 and AL237 are smaller than the phenoxyacetamide-based inhibitors and thus cannot fully span the gorge. Instead, these inhibitors adopted different binding poses in *Ag*AChE1, each corresponding to a distinct cluster (Figure 9C,D). The stronger inhibitor AL201 produced three different conformational interaction states while the weaker AL237 had two.

**Figure 10:**
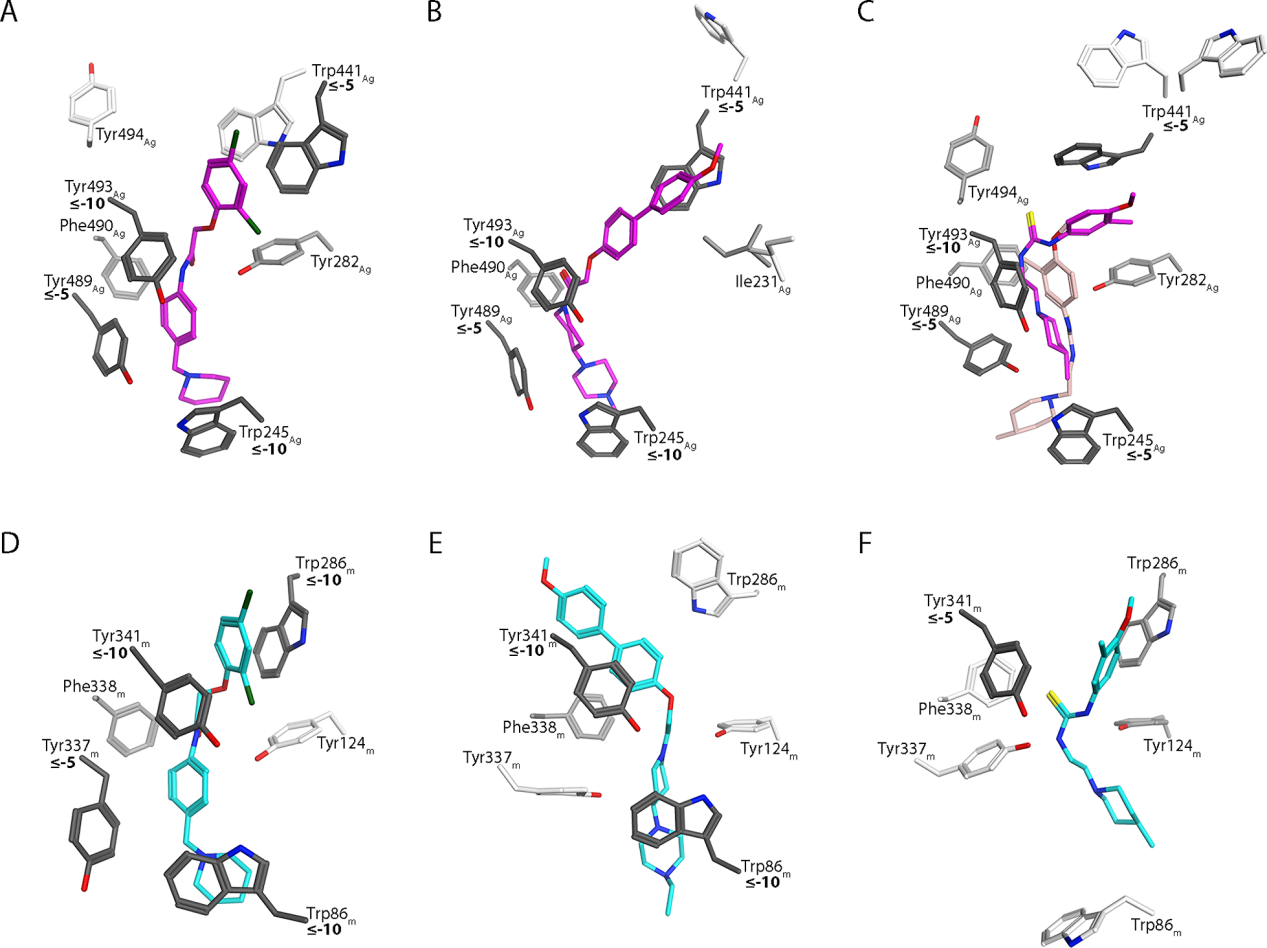
Illustration of the binding energy contributions of AChE amino acid residues and the inhibitors showing strong inhibition for *Ag*AChE1 (IC_50_ < 1 μM). (A–C) Show AL137, AL284 and AL201 interacting with *Ag*AChE1 and (D–F) show AL137, AL284 and AL201 interacting with *m*AChE. The interaction energies were calculated using the MM-PBSA method. Interaction energy values are shown in bold numbers in kJ/mol.

The binding energy contributions of individual amino acid residues and inhibitors were calculated using the MM-PBSA method^53^ for representative conformers of each cluster (Figures S26, S27 and 10). For AL137 and AL284 in complex with *Ag*AChE1, all clusters featured strong interactions between the inhibitor and Trp245_Ag_ and Tyr493_Ag_ at the bottom and middle of the gorge, respectively (≤−10 kJ/mol; Figure 10). In addition, AL137 and AL284 had favorable interactions with Tyr489_Ag_ in the α-helix next to loop 2, and Trp441_Ag_ at the top of the gorge (≤−5 kJ/mol). The interaction strength with Trp441_Ag_ was weakened in some binding poses. The representative conformers of the many clusters of *Ag*AChE1•AL264 had weaker interactions than the conformers of the other phenoxyacetamide-based inhibitors, but the total interaction energies between each residue and AL264 over all clusters were comparable in strength to those for AL137 and AL284 (Figures S26).

All binding poses of AL201 in complex with *Ag*AChE1 had strong interactions with Tyr493 (≤−10 kJ/mol), and strong or favorable interactions with Trp245 (≤−5 kJ/mol) at the bottom of the gorge, thus adopting a similar binding pattern to the phenoxyacetamide-based inhibitors. The different binding poses were also characterized by differing interaction strengths: the largest cluster had a stronger interaction with Trp441_Ag_ in loop 1 at the top of the gorge, the second largest had a stronger interaction with Trp245 at the bottom (binding lower down in the gorge), while the third had a favorable interaction with Tyr494_Ag_ in loop 2 (see Figures 9D and 10C). In addition, all three clusters for AL201 featured favorable inhibitor interactions with Tyr489_Ag_ in the α-helix next to loop 2, like the potent inhibitors AL137 and AL284. The weak inhibitor AL237 produced two markedly different binding poses (Figure 9E). The dominant conformational interaction state (67%) had favorable interactions with Trp245_Ag_ and Tyr489_Ag_ in the lower part of the gorge, while second bound higher up in the gorge forming strong interactions with Tyr493_Ag_ and favorable interactions with Tyr282_Ag_, Trp441_Ag_, and Phe449_Ag_ (Figure S26).

The cluster analysis of *m*AChE in complex with the potent phenoxyacetamide-based inhibitor AL137 revealed four conformational interaction states with the dominant state accounting for 54% of the total conformations (Table S14). Binding energy calculations revealed that this dominant cluster had strong interactions between AL137 and Trp86_m_ at the bottom of the gorge and with Trp286_m_ and Tyr341_m_ at the top (Figures 10D and S27). The inhibitor also formed favorable interactions with Tyr337_m_ and Phe338_m_ in the α-helix next to loop 2 of *m*AChE. The two main conformational interaction states of the AL284 complexes with *m*AChE featured strong inhibitor interactions with Trp86_m_ and Tyr341_m_ but not with Trp286_m_, leading to a significantly weaker overall interaction with Trp286_m_ than for AL137 (Figures 10E and S27). Binding energy calculations of the binding poses of the three other inhibitors (AL264, AL201 and AL237) revealed few strong interactions with the amino acid residues in the gorge of *m*AChE (Figures S27 and 10F).

In summary, the strong inhibitors formed favorable to strong interactions with at least four amino acid residues flanking the active site gorge. For the smaller AL201, this was achieved by adopting multiple binding poses in *Ag*AChE1. The weaker inhibitors had similar interaction patterns to the strong inhibitors but had fewer favorable to strong interactions. The main difference between *Ag*AChE1 and *m*AChE in terms of the interaction patterns of the selective inhibitors AL284 and AL201 related to the interactions formed between the aromatic moieties of Tyr489_Ag_ and Phe490_Ag_ in the α-helix and the aliphatic amines of AL284 and AL201. The altered positioning of the α-helix of *m*AChE compared to *Ag*AChE1 modified the shape of the gorge (see above), preventing the formation of this interaction with AL284 and AL201. The nonselective AL137 instead forms favorable arene–arene interactions with Tyr489_Ag_ in *Ag*AChE1 and Tyr337_m_ in *m*AChE.

## Conclusion

Despite their overall structural and amino acid sequence similarities, there are profound differences in the MD of *Ag*AChE1 and *m*AChE: *m*AChE is more dynamic and the three loops flanking the active site gorge, which participate in the largest collective movements, differed in their dynamic patterns. We can conclude that the proline sequence differences between *Ag*AChE1 and *m*AChE in these three loops profoundly affect the nature and character of the enzyme’s main chain dynamics. It is reasonable to assume that the stronger dynamics observed in *m*AChE as an effect of proline residue mutations have evolved over time since the vertebrate nervous system is more refined than that of insects. The fact that AChEs from vertebrates hydrolyze acetylcholine more rapidly than AChEs from insects may thus be due to their more pronounced dynamics and their more open entrance conformation, which would facilitate rapid entry of the substrate to the active site while also accelerating product ejection.

When compared to weak inhibitors, strong inhibitors of *Ag*AChE1 had more favorable interactions with aromatic residues in the active site gorge and stabilized the Ω loop without inducing fluctuations of loop 2. We conclude that this explains the differing potency profiles of these inhibitors. Interestingly, rather than single binding poses, 2–4 poses were detected for each strong inhibitor. These results highlight the importance of considering dynamics when determining structure activity relationships for AChE-systems. Furthermore, multiple long MD simulations with careful sampling were strictly required to obtain these results because it was necessary to thoroughly characterize the movements of the three loops at the gorge entrance in order to understand the AChE•inhibitor interaction patterns. The selectivity profiles of the inhibitors could be linked to dynamic differences in the active site gorge of the two enzymes. The shape of the gorge depended on the dynamics of the three loops, which differed between *Ag*AChE1 and *m*AChE. Importantly, the aliphatic amines of the selective inhibitors formed strong interactions with Tyr489_Ag_ of *Ag*AChE1 that were absent in *m*AChE. The water analysis showed that the stronger inhibitors had on average more water molecules close to the hydroxyl oxygen of Tyr282_Ag_/Tyr124_m_ compared to the weak inhibitors, although still decreased compared to apo enzymes. These results may indicate that water transit is important for obtaining potent AChE inhibitors, and warrants further investigations of water passages and the free energy change for exchanging water with the bulk upon ligand binding.

AChE is a validated insecticidal target for mosquito-borne diseases but current insecticidal inhibitors of this enzyme have important limitations resulting from safety concerns and resistance. Noncovalent inhibitors specifically targeting AChE1 offer new potential mechanisms of action compared to those in current use that target the conserved active site. Our study shows that there are large differences between the dynamic patterns of *Ag*AChE1 and *m*AChE, and that the potency and selectivity profiles of such closely related enzymes can be determined by studying the dynamics of AChE•inhibitor complexes. Our results thus offer a rational computational approach for discovering improved insecticides.

## Material and Methods

### IC_50_ Determination

Dose–response curves for AL201 and AL237 were determined for *m*AChE using 100 mM DMSO stock solutions of the inhibitors. Dilutions for the measurements were prepared in Milli-Q water for AL201 and sodium phosphate buffer (0.1 M, pH 7.4) for AL237 due to solubility issues. Dilution series of eight concentrations were prepared (0.001–1000 and 0.1–1000 μM, respectively). Enzymatic activity was measured using the Ellman assay (adapted to a 96-well format) with secreted nonpurified protein in growth medium. A QIAgility robotic benchtop instrument (Qiagen) was used for automatic liquid handling when setting up assay plates. The assay was then performed at 30 °C in a final volume of 200 mL 0.1 M phosphate buffer (pH 7.4) containing 0.2 mM 5,5′-thiobis(2-nitrobenzoic acid) and 1 mM acetylcholine iodide. Changes in the wells’ absorbance at 412 nm were recorded over 60 s using a FlexStation 3 Multi-Mode Microplate Reader (Molecular Devices) to monitor the enzymatic reaction. Eight positive controls of uninhibited enzyme were assumed to represent 100% activity and used to determine the average slope. Activity observed in wells with inhibitors was quantified in relation to this value. Nonlinear regression (curve fitting) in GraphPad Prism^54^ was used to calculate IC_50_ values. The log[inhibitor] *vs* response variable slope equation was fitted using three parameters. Three independent replicates were used for the IC_50_ determination. IC_50_ data for AL201 and AL237,^21^ AL284 and AL264,^22^ AL137^20,26^ were taken from previous publications.

### X-ray Crystallography

Wild-type *m*AChE from *Mus musculus* was expressed in HEK293F cells and purified and crystallized as previously described.^55^ Briefly, HEK293F cells expressing secreted *m*AChE were grown in suspension using Freestyle 293 and Glutamax (Gibco) media containing 20 μg mL^–1^ Gentamicin (Gibco). The *m*AChE-containing supernatant was centrifuged and the cleared supernatant was purified using affinity and size exclusion chromatography. Protein crystallization was done by the hanging drop vapor diffusion method at a protein concentration of 10 mg mL^–1^ with a well solution containing 27–30% (w/v) PEG750MME and 0.1 M HEPES, pH 6.9–7.1. The crystals were grown at a temperature of 277 K. The complexes were generated by soaking the inhibitors into *m*AChE crystals before flash freezing in liquid nitrogen as previously described. In short, to generate a complex between AL237 and *m*AChE, several grains of AL237 was dissolved in 30% PEG750 MME, 100 mM HEPES pH 7.1 to generate a soak stock solution. Following a brief sonication, several 1 μL portions of the soak stock solution was added to a drop that contained several crystals of *m*AChE. After an incubation of 5–10 min, diffraction data was collected. X-ray data collection was performed at the MAX-lab synchrotron and on the MAXIV synchrotron using the BioMAX beamline (Lund, Sweden). Data processing and refinement of *m*AChE•AL237 was performed using the same software, rejection criteria, and strategy as applied for the previously reported crystal structures included in this study.^50^ The intensity data were indexed and integrated by XDS and scaled using Aimless in the CCP4 suite. The structure was determined by difference Fourier methods with a modified apo structure of AChE (PDB code 1J06) as a starting model, using Refmac. Further crystallographic refinement and manual rebuilding were performed using Phenix^56^ and COOT.^57^ The structure was refined using an occupancy of 1.0 for the AL237 ligand. The model’s quality was validated using MolProbity (in Phenix^56^) and the wwPDB Validation Service. Due to the symmetry of the AL201 molecule and the disordered electron density observed in multiple collected X-ray data sets, a model of *m*AChE•AL201 could not be obtained with satisfying validation. Data collection and refinement statistics for *m*AChE•AL237 (Figure S13) are listed in Table S4. The coordinates and structure factors have been deposited in the RCSB Protein Data Bank with accession code 8ORC.

### Molecular Dynamics Simulations

X-ray crystal structures of apo *Ag*AChE1 (PDB: 5X61), apo *m*AChE (PDB: 1J06), *m*AChE in complex with inhibitors AL284, AL264 and AL137 (PDB codes: 6FSE, 6FSD, and 5FOQ, respectively), and the new structure obtained in this work were used as the starting coordinates for the MD simulations. The missing coordinates for residues 258–264 of the apo *m*AChE crystal structure (Figure S1) were modeled using Modeler 9.18.^58^ AL201 was modeled *in silico* into the binding site of *m*AChE, taking the experimentally determined electron density map into account. To obtain *Ag*AChE1•inibitor complexes the coordinates of the inhibitors were manually copied from the *m*AChE•inhibitor complexes and superimposed on the apo *Ag*AChE1 structure (PDB: 5X61) using PyMol.^59^

### Inhibitor Protonation States

The coordinates of the inhibitors were extracted from the complexes and hydrogen atoms were added using the Open-Babel package with an assumed pH of 7.4.^60^ This resulted in protonation of the following atoms: the ethyl-substituted nitrogen of the piperazine fragment of AL284, the piperidine nitrogens of AL137, AL264 and AL201, and the dimethylamine nitrogen of AL237.

### Inhibitor Force-Field Parameters

Electrostatic surface potentials of the inhibitors were calculated after geometry optimization using the HF/6-31G* basis set with Gaussian 09.^61^ The ESPs were then used to calculate partial atomic charges using the restrained electrostatic potential (RESP) method with the antechamber program of AmberTools.^62^ All other parameters were assigned from the General Amber Force Field. AMBER topology/coordinate files were created using the AmberTools parmchk and tleap programs. These files were converted from AMBER to GROMACS format using the acpype python script.^63^

### Molecular System Setup

The pdb 2gmx within the GROMACS package^64^ was used to generate topology and coordinate files for *Ag*AChE1 and *m*AChE using the AMBER99SB-ILDN force field. The resulting topology and coordinates files were merged with those of the inhibitors to obtain files for the *Ag*AChE1•inhibitor and *m*AChE•inhibitor complexes. The molecular systems were positioned at the center of a dodecahedral periodic box, solvated by adding water molecules, and neutralized by adding counterions with an excess of 0.150 M NaCl to mimic a physiological ion concentration. The TIP3P parameters were used for water molecules during the simulations.

### MD Setup

MD simulations were performed using the GROMACS 5.1.2 simulation package.^64^ The energies of the molecular systems were minimized using the steepest descent algorithm to remove atomic clashes. The systems were heated from 0 to 300 K over 100 ps NVT simulations, followed by equilibration of densities at 1 atm pressure over 500 ps NPT simulations. In these simulations, all heavy atoms were restrained at the starting positions with a force constant of 1000 kJ mol^–1^ nm^–2^. These restraints were linearly removed during a subsequent 1 ns NPT simulation. The temperature and pressure were regulated using the Berendsen algorithm. Subsequently, 5*500 ns and 3*200 ns equilibrium simulations with random initial velocities were performed for the apo *Ag*AChE1, apo *m*AChE, *Ag*AChE1•inhibitor, and *m*AChE•inhibitor complexes. The temperature and pressure were maintained at 300 K and 1 atm with 0.1 and 1 ps time constants using the v-rescale temperature and the Parrinello–Rahman pressure coupling method, respectively. The short-range nonbonded interactions were computed for atom pairs within a 14 Å distance. Long-range electrostatic interactions were calculated using the Particle-Mesh-Ewald summation method with fourth-order cubic interpolation and a 1.2 Å grid spacing. The time-steps during the simulations were 2 fs with all bonds constrained using the parallel LINCS algorithm.

### MD Trajectory Analysis

rmsd values for simulated coordinates of *Ag*AChE1, *m*AChE, or inhibitors were calculated after superimposing main chain atoms on the NPT simulated starting structure as a reference using the gmx rms module in GROMACS. Based on the rmsd values, the first 50 ns from each trajectory was discarded and the remaining trajectories were concatenated and used for all subsequent analyses. Root-mean-square fluctuation (RMSF) values were calculated after superimposing main chain atoms on the NPT simulated starting structure as a reference using the gmx rmsf module in GROMACS.

### PCA and Conformational Clustering of Apo *Ag*AChE1 and *m*AChE

The mass-weighted covariance matrix of the superimposed main chain coordinates of the NPT simulated starting structure was calculated, and eigenvectors with their respective eigenvalues were obtained using gmx covar. Subsequently, projections of the trajectory on the first three largest eigenvectors *i.e.*, PCs, were calculated with gmx anaeig. Similarly, projections of energy minimized X-ray crystal structures were also calculated for comparison with the MD simulations. To visualize the motions along a PC, 100 conformations were interpolated between two extremes of the PC and dumped as the trajectories by gmx anaeig. Local conformational ensembles of the PCA subspace were determined through cluster analysis using gmx_clusterByFeatures^65^ with the first three PCs as the features. For clustering, the K-means algorithm was used and the number of clusters were determined using the Elbow method with a threshold of 2.5% applied on the sum of square residual to sum of square total ratio. The PC1 values for apo *m*AChE were transformed by multiplication with −1, for visualization purposes when comparing with apo *Ag*AChE1.

### PCAs of *Ag*AChE1•Inhibitor and *m*AChE•Inhibitor Complexes

The main chain atoms extracted from the trajectories of the apo and inhibited *Ag*AChE1 and *m*AChE, respectively, were concatenated into two single trajectories, one for each species. PCAs were performed on each trajectory as described in the section above. The projections of the apo and inhibited enzyme trajectories onto the first three largest eigenvectors were then calculated for *Ag*AChE1 and *m*AChE. Additionally, the overlap of these PCs with the corresponding PCs from the apo forms (as obtained in the above section) were computed using gmx anaeig.

### Minimum Distances

The gmx pairdist module in GROMACS was used to calculate the pairwise minimum distances between either main chain or all heavy atoms of selected residues.

### Water Analysis

The gmx trjorder module in GROMACS was used for calculating the number of water molecules that were positioned at hydrogen bonding distances of selected amino acid residue atoms in the gorge of AChE over the trajectory times. Water occupancy at heavy atom distances of <3.5 Å or <3.0 Å to residue atoms of AChE was then calculated as averaged number of water molecules over the MD trajectories (50–500 ns and 50–200 ns for apo AChE and AChE•inhibitor complexes, respectively). The amino acid residue atoms were selected based on conserved waters in the binding gorge of crystal structures of *m*AChE and *h*AChE (Tables S7–S9 and Figure S22). The water analysis was made on crystal structures previously extracted from the RCSB Protein Data Base with the criteria of a resolution of ≤2.3 Å,^24^ and complemented with *m*AChE•AL237 (PDB: 8ORC).

### *Ag*AChE1•Inhibitor and *m*AChE•Inhibitor Clustering based on Dynamics and Interaction Pattern

The widely used rmsd matrix-based clustering method fails to capture changes in the binding interaction networks among clusters, particularly when both the protein and the inhibitors are flexible. Additionally, this method is costly in terms of both memory and time for long trajectories. To overcome these limitations, we developed a fast method that captures both inhibitor dynamics and binding interaction networks among clusters effectively from long MD trajectories. The method uses a feature-based clustering approach implemented in gmx_clusterByFeatures. First, the reciprocals of the minimum distances were calculated between all pairs of *Ag*AChE1 or *m*AChE residues and inhibitor fragments for each frame in the trajectory. By reciprocating the distance, the magnitude of distant residue fluctuations was reduced while that for residues neighboring the inhibitors was increased. In the next step these distances, which were stored in place of coordinates in a trajectory file, were used to calculate the eigenvectors and eigenvalues using gmx covar. Subsequently, projections of distances on the first three largest eigenvectors were computed using gmx anaeig. These three PCs were used as the features to perform clustering using the K-means method with gmx clusterByFeatures. The number of clusters was determined on the basis of a Silhouette score and a Davies-Bouldin index in combination with features and structure visualization. The obtained clusters have either the lowest or second lowest Davies-Bouldin index. The central structure and the first 1000 frames most similar to the central structure in feature-space were extracted from each cluster for further analysis.

### Evaluation of *Ag*AChE1•Inhibitor and *m*AChE•Inhibitor Interaction Energies

To evaluate the *Ag*AChE1•inhibitor and *m*AChE•inhibitor interaction strengths for conformers in each cluster obtained in the above section, the binding energies were calculated using the MM/PBSA method with g_mmpbsa.^53^ For the energy calculations, the 20 frames (conformers) closest to the central structure of each cluster, evenly spaced by 20 frames, were taken to represent local free energy minima in the feature subspace. The resulting binding energies were decomposed per residue to determine their contribution to inhibitor binding. Noncharged residues with energy contributions less than or equal to −4 kJ/mol were considered for further analysis.

### Gorge Radius Calculation

Gorge radii were calculated with the HOLE software^66^ using the hole subcommand included in gmx_clusterByFeatures.^65^ The NPT simulated starting structure was taken as a reference and reoriented using Chimera^67^ such that the gorge axis became parallel to the *Y*-axis. Next, all conformers in the MD trajectory were superimposed onto the reference structure by least-squares fitting of C-alpha atoms and saved as a new trajectory. The hydrogen atom on the α-carbon of Gly122 was considered as the seed coordinate for radius calculation since this atom remained exposed at the gorge surface throughout the simulations. Furthermore, the gorge axis was divided into 0.5 Å thick slices and the radius was determined over 2000 Monte Carlo steps for each slice. Finally, we obtained the radius of each slice for each frame in the trajectory. To visualize the radius of the entire gorge, a range of 2.5–20 Å along the gorge axis was considered and the radius distributions were plotted. In addition, the position distribution was plotted for each residue present at the gorge surface. These plots were generated using holeplot from gmx_clusterByFeatures.

## Supporting Information Available

Sequence alignment of parts of *Ag*AChE1 and *m*AChE; rmsd-plots of MD simulations of apo *Ag*AChE1 and *m*AChE; eigenvalues and distances for motions of apo *Ag*AChE1 and *m*AChE; cluster analysis of apo *Ag*AChE1 and *m*AChE; gorge radius calculations; crystal structure of *m*AChE•AL237; LigPlot diagrams of *m*AChE•inhibitor complexes; rmsd-plots of MD simulations of inhibited *Ag*AChE1 and *m*AChE; investigation of largest collective motions for AChE•inhibitor complexes; the effect of inhibitors on local enzyme dynamics; waters in the active site gorges of *Ag*AChE1 and *m*AChE; interactions between inhibitors and enzymes (PDF)
