# Supplementary material for: Enzyme Dynamics Determine the Potency and Selectivity of Inhibitors Targeting Disease-Transmitting Mosquitoes
Source: ACS Infect Dis. 2024 Sep 18;10(10):3664–80. doi: 10.1021/acsinfecdis.4c00531 (PMC11474975; doi:10.1021/acsinfecdis.4c00531)
Supplement: Supplementary file 1 — id4c00531_si_001.pdf [file id4c00531_si_001.pdf]

SUPPORTING INFORMATION

Enzyme Dynamics Determine the Potency and Selectivity of Inhibitors Targeting Disease-Transmitting Mosquitoes

Rashmi Kumari,[a‡] Cecilia Lindgren,[a‡] Rajendra Kumar,[a] Nina Forsgren,[b] C. David Andersson,[a] Fredrik Ekström,[b] Anna Linusson[a*]

[a]*Department of Chemistry, Umeå University, SE-90187, Umeå, Sweden.* [b]*CBRN Defense and Security, Swedish Defense Research Agency, SE-90621, Umeå Sweden*

**E-mail: anna.linusson@umu.se*

[‡]*R.K. and C.L. contributed equally to this paper*

Table of Contents

Sequence alignment of selected parts of *Ag*AChE1 and *m*AChE S2

RMSD-plots of MD simulations of apo *Ag*AChE1 and *m*AChE S3

Eigenvalues and distances for motions of apo *Ag*AChE1 and *m*AChE S4

Cluster analysis of apo *Ag*AChE1 and *m*AChE S6

Gorge radius calculations S9

Crystal structure of *m*AChE•AL237 .... S13

LigPlot diagrams of *m*AChE•inhibitor complexes S15

RMSD-plots of MD simulations of inhibited *Ag*AChE1 and *m*AChE S16

Investigation of largest collective motions for AChE•inhibitor complexes S20

The effect of inhibitors on local enzyme dynamics S21

Waters in the active site gorges of *Ag*AChE1 and *m*AChE S23

Interactions between inhibitors and enzymes S29

References S33

Sequence alignment of selected parts of *Ag*AChE1 and *m*AChE

Table S1. Sequence alignment of the Ω loop for *Ag*AChE1 and *m*AChE

Species	Sequence																										
	loop region 1													α-helix region					loop region 2									
*Ag*AChE1	Cys228	Val229	Gln230	Ile231	Val232	Asp233	Thr234	Val235	Phe236	Gly237	Asp238	Phe239	Pro240	Gly241	Ala242	Thr243	Met244	Trp245	Asn246	Pro247	Asn248	Thr249	Pro250	Leu251	Ser252	Glu253	Asp254	Cys255
*m*AChE	Cys69	Tyr70	Gln71	Tyr72	Val73	Asp74	Thr75	Leu76	Tyr77	Pro78	Gly79	Phe80	Glu81	Gly82	Thr83	Glu84	Met85	Trp86	Asn87	Pro88	Asn89	Arg90	Glu91	Leu92	Ser93	Glu94	Asp95	Cys96

Table S2. Sequence alignment of loop 1 for *Ag*AChE1 and *m*AChE

Species	Sequence														
*Ag*AChE1	Asn439	Glu440	Trp441	Gly442	Thr443	Leu444	-[a]	-	-	Gly445	Ile446	Cys447	Glu448	Phe449	Pro450
*m*AChE	His284	Glu285	Trp286	His287	Val288	Leu289	Pro290	Gln291	Glu292	Ser293	Ile294	Phe295	Arg296	Phe297	Ser298

[a] – defines a gap in the sequence alignment between *Ag*AChE1 and *m*AChE

Table S3. Sequence alignment of the α-helix/loop 2 for *Ag*AChE1 and *m*AChE

Species	Sequence																						
	α-helix region									loop region														
*Ag*AChE1	Glu485	Glu486	Gly487	Tyr488	Tyr489	Phe490	Ile491	Ile492	Tyr493	Tyr494	Leu495	Thr496	Glu497	Leu498	Leu499	Arg500	Lys501	Glu502	Glu503	Gly504	Val505	Thr506	Val507	Thr508
*m*AChE	Asp333	Glu334	Gly335	Ser336	Tyr337	Phe338	Leu339	Val340	Tyr341	Gly342	Val343	-[a]	Pro344	Gly345	Phe346	Ser347	Lys348	Asp349	Asn350	Glu351	Ser352	Leu353	Ile354	Ser355

[a] – defines a gap in the sequence alignment between *Ag*AChE1 and *m*AChE

RMSD-plots of MD simulations of apo *Ag*AChE1 and *m*AChE

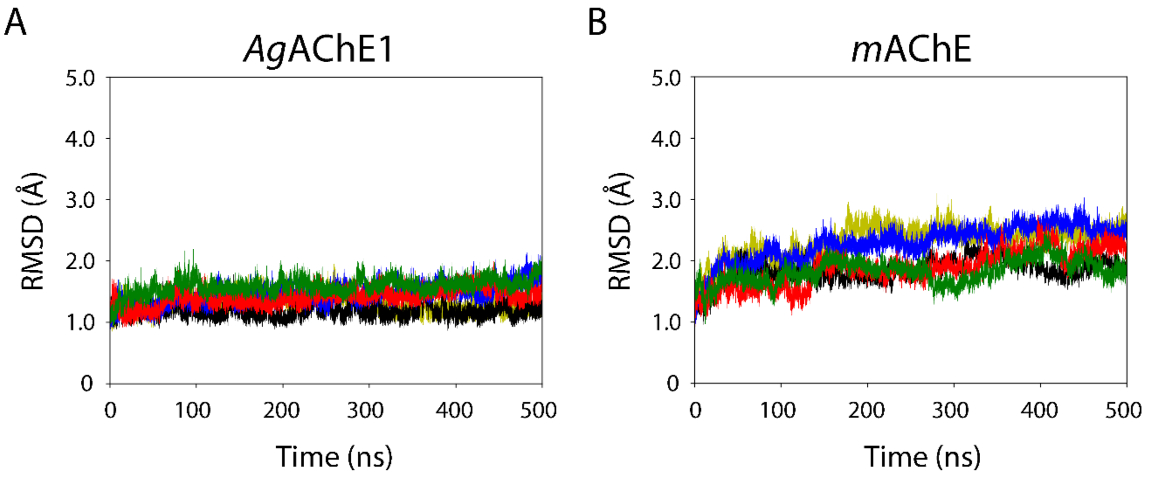

Figure S1. Root mean square deviation (RMSD) values of *Ag*AChE1 (**A**) and *m*AChE (**B**) protein backbone vs simulation time for the five separate MD simulations.

Eigenvalues and distances for motions of apo *Ag*AChE1 and *m*AChE

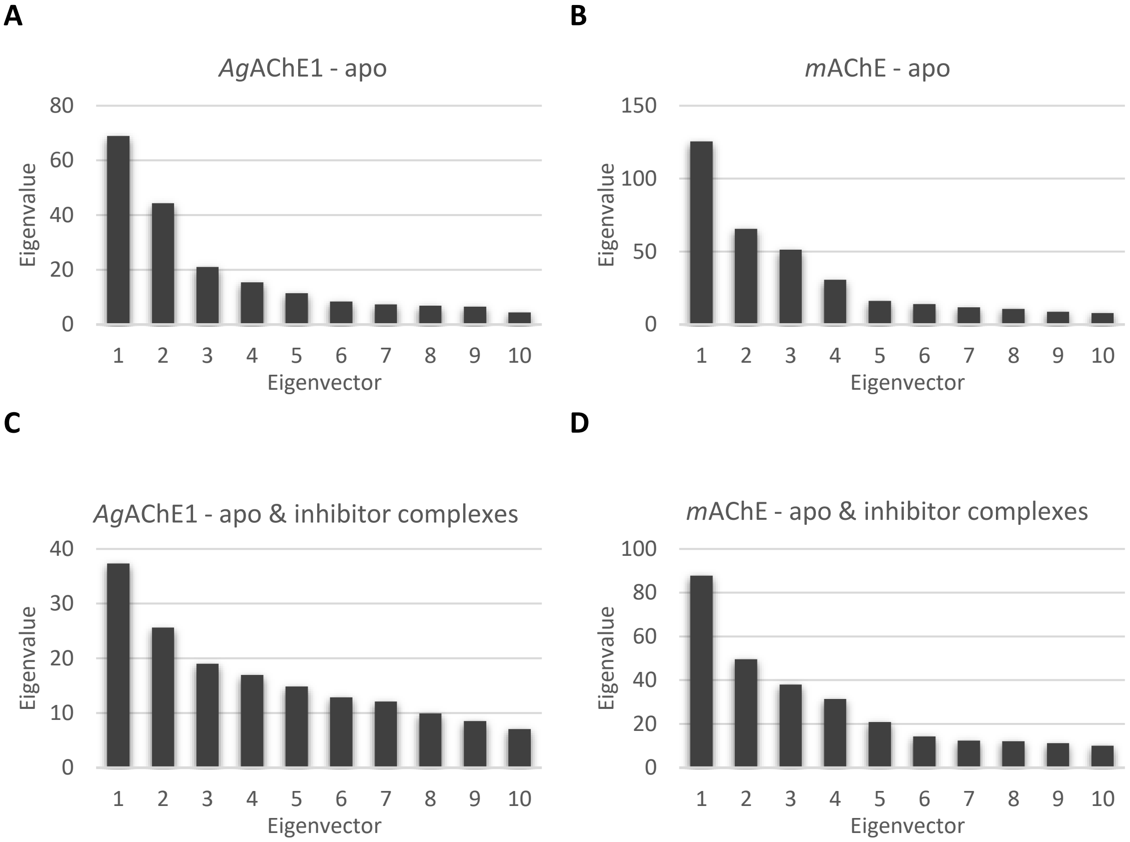

Figure S2. Principle component analysis (PCA) was used to investigate the largest collective motions of the enzymes. The PCA was performed on the protein main chain atoms of the combined MD simulations of *Ag*AChE1 and *m*AChE, respectively. The higher eigenvalues of the PCs of *m*AChE compared to *Ag*AChE1 show that *m*AChE was more dynamic than *Ag*AChE1. The eigenvalues of the first ten PCs (eigenvectors) over combined simulations are shown for **A**) apo *Ag*AChE1, **B)** apo *m*AChE, **C)** apo *Ag*ChE1 and the five *Ag*AChE1•inhibitor complexes, and **D)** apo *m*AChE and the five *m*AChE•inhibitor complexes.

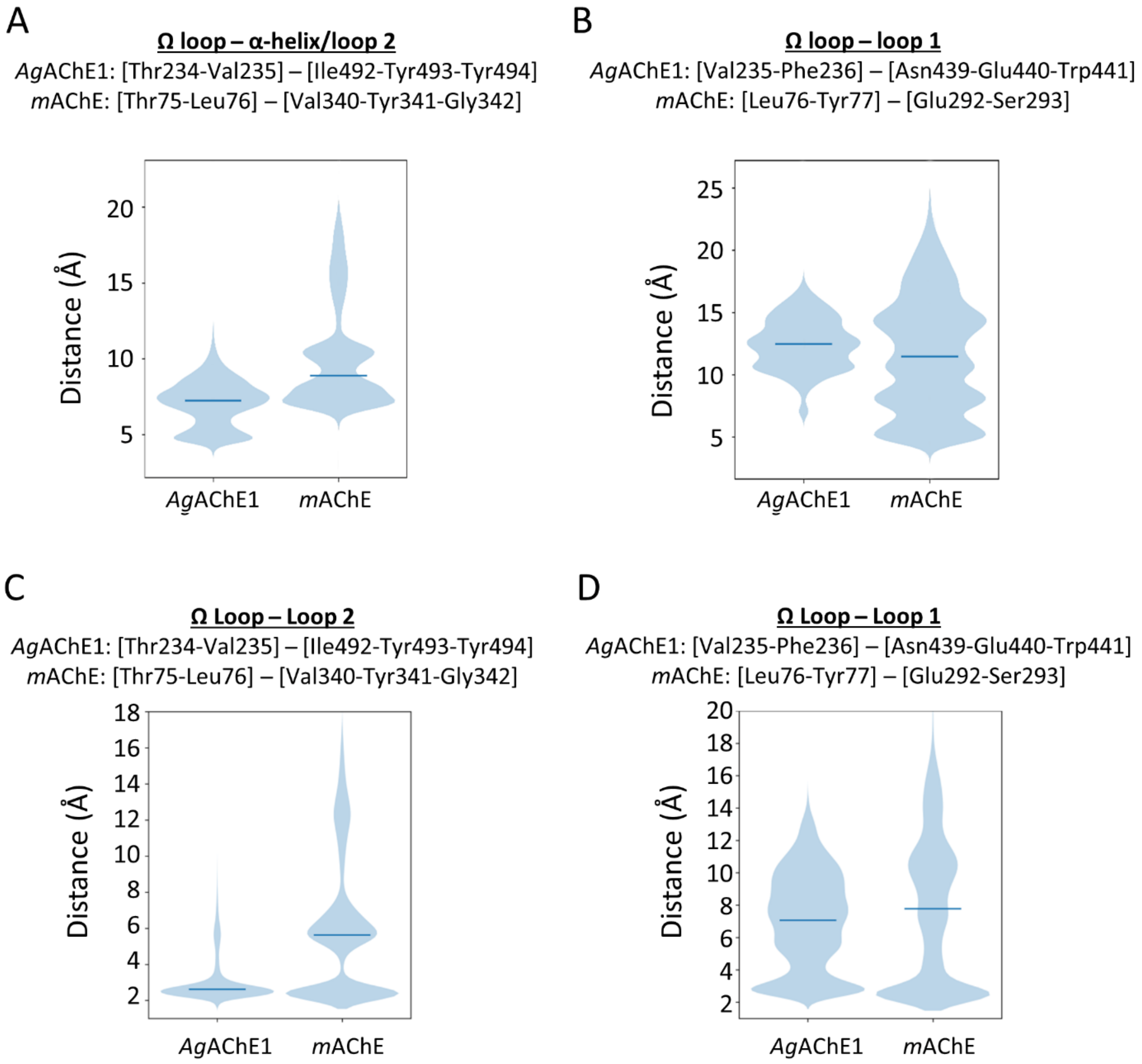

Figure S3. Monitoring of the size of the gorge entrance for apo *Ag*AChE1 compared to apo *m*AChE. The measured distances verify that the gorge entrance is significantly wider for *m*AChE. Pairwise minimum distances between the entrance loops are calculated as the minimum distances between main chain atoms or heavy atoms of selected residues in the loops comparing *Ag*AChE1 and *m*AChE. **A)** Minimum distance between the main chain atoms of the Ω loop and α-helix/loop 2. **B)** Minimum distance between the main chain atoms of the Ω loop and loop 1. **C)** Minimum distance between the heavy atoms of the Ω loop and α-helix/loop 2. **D)** Minimum distance between the heavy atoms of the Ω loop and loop 1. The distances were calculated for the original trajectory (50-500ns). The dark blue line represents the mean value of the minimum distance throughout the MD simulation, and the width of the light blue shape represents the fraction of frames at each distance.

Cluster analysis of apo *Ag*AChE1 and *m*AChE

Table S4. Clusters of ensembles of local conformational states obtained from cluster analysis based on the first three PCs of the PCA subspace of MD simulations of apo *Ag*AChE1. The number of frames as well as the occurrence for each cluster are reported.

Cluster ID	Number of Frames	Occurrence (%)
1	89323	40
2	45825	20
3	45002	20
4	44851	20

Table S5. Clusters of ensembles of local conformational states obtained from cluster analysis based on the first three PCs of the PCA subspace of MD simulations of apo *m*AChE. The number of frames as well as the occurrence for each cluster are reported.

Cluster ID	Number of Frames	Occurrence (%)
1	52044	23
2	44994	20
3	42960	19
4	42519	19
5	42484	19

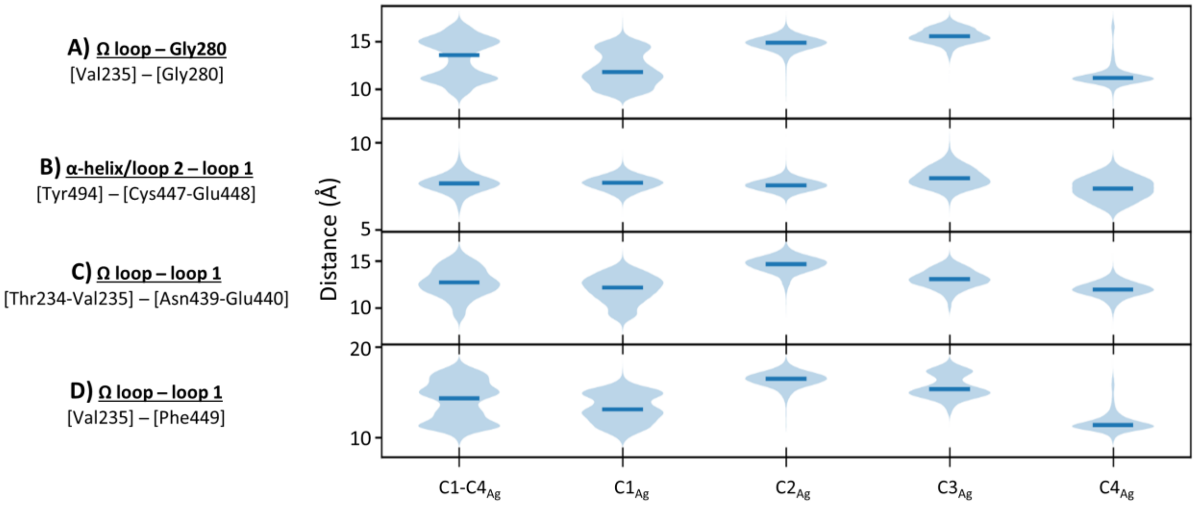

Figure S4. Pairwise minimum distances between main chain atoms in the entrance loops of the resulting clusters (conformational states) of apo *Ag*AChE1. The intermediate states ($C1_{Ag}$ and $C2_{Ag}$) differs compared to the open ($C3_{Ag}$) and closed ($C4_{Ag}$) state, shown by the opening/closing of the gorge performed by **A)** the Ω loop in relation to Gly280 and **B)** the α-helix/loop 2 in relation to loop 1. **C)** and **D)** the difference between the intermediate states ($C1_{Ag}$ and $C2_{Ag}$) shown by distances between the Ω loop and loop 1. The distances were calculated for the conformations of the original MD trajectory (50-500 ns). The dark blue line represents the mean value of the minimum distance throughout the MD simulation, and the width of the light blue shape represents the fraction of frames at each distance.

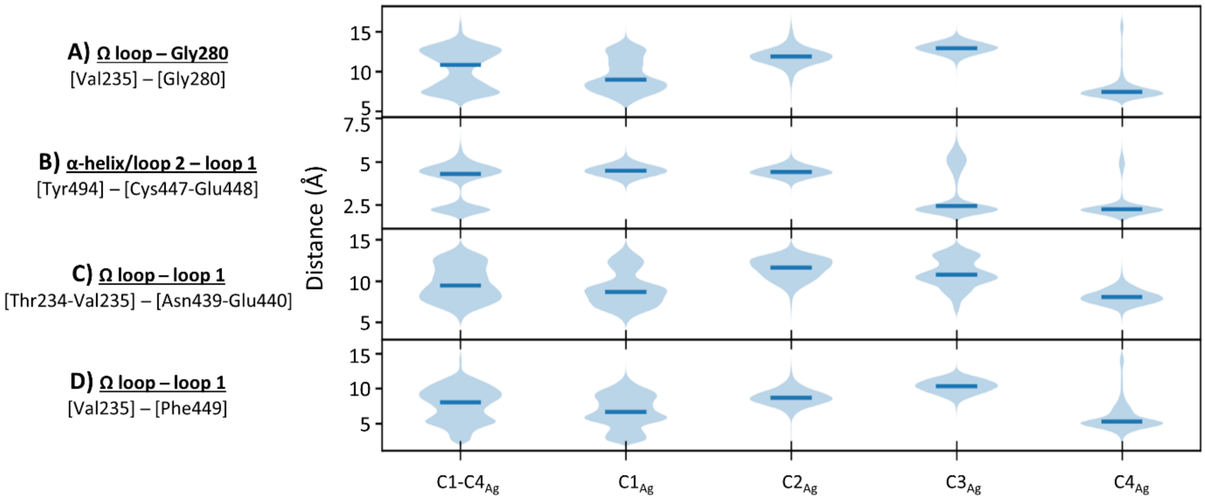

Figure S5. The pairwise minimum distances were calculated as the minimum distances between all atoms of selected residues in the loops, as extension of Figure S4. **A)** The Ω loop in relation to Gly280. **B)** The α-helix/loop 2 in relation to loop 1. **C)** and **D)** the Ω loop in relation to loop 1. The distances were calculated for the conformations of the original MD trajectory (50-500 ns) divided into the clusters obtained from the cluster analysis of the first three PCs of the PCA subspace of *Ag*AChE1 to visualize the differences between the ensembles of local conformational states. The dark blue line represents the mean value of the minimum distance throughout the MD simulation, and the width of the light blue shape represents the fraction of frames at each distance.

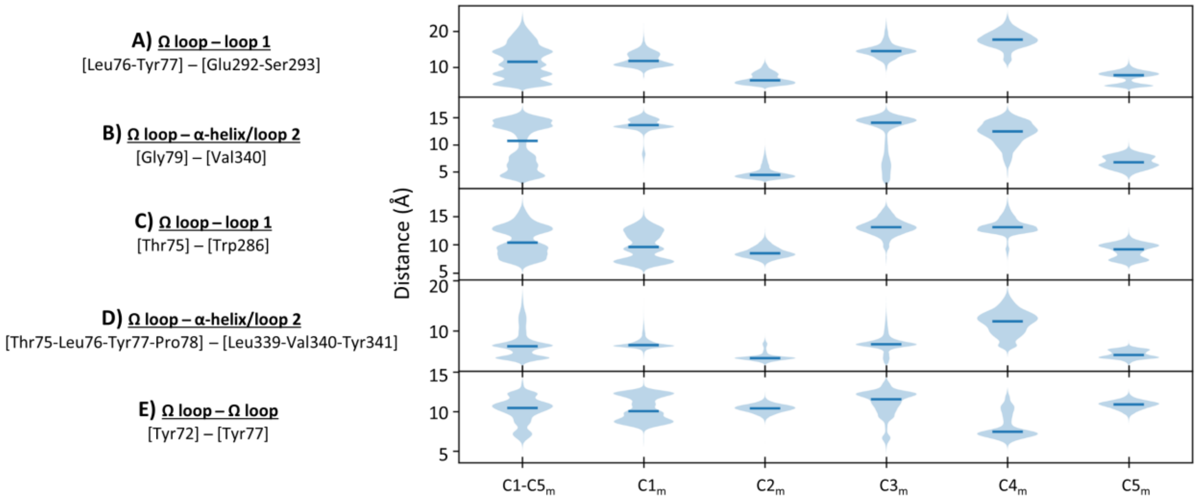

Figure S6. Pairwise minimum distances between main chain atoms in the entrance loops of the resulting clusters (conformational states) of apo *m*AChE1. **A)** The difference between the open ($C1_m$. $C3_m$ and $C4_m$) and closed ($C2_m$ and $C5_m$) states, shown by the opening/closing of the gorge performed by the Ω loop in relation to loop 1. **B)** The difference between the open ($C1_m$. $C3_m$ and $C4_m$) and closed ($C2_m$ and $C5_m$) states shown by the Ω loop in relation to the α-helix/loop 2. **C)** The difference between the open state $C1_m$ and the other two ($C3_m$ and $C4_m$) shown by the displacement of the Ω loop in relation to loop 1. **D)** The difference between the open states $C3_m$ and $C4_m$, shown by the positioning of the Ω loop in relation to α-helix/loop 2. **E)** The difference between the open states $C3_m$ and $C4_m$, shown by the rearrangement of the Ω loop. The distances were calculated for the conformations of the original MD trajectory (50-500 ns). The dark blue line represents the mean value of the minimum

distance throughout the MD simulation, and the width of the light blue shape represents the fraction of frames at each distance.

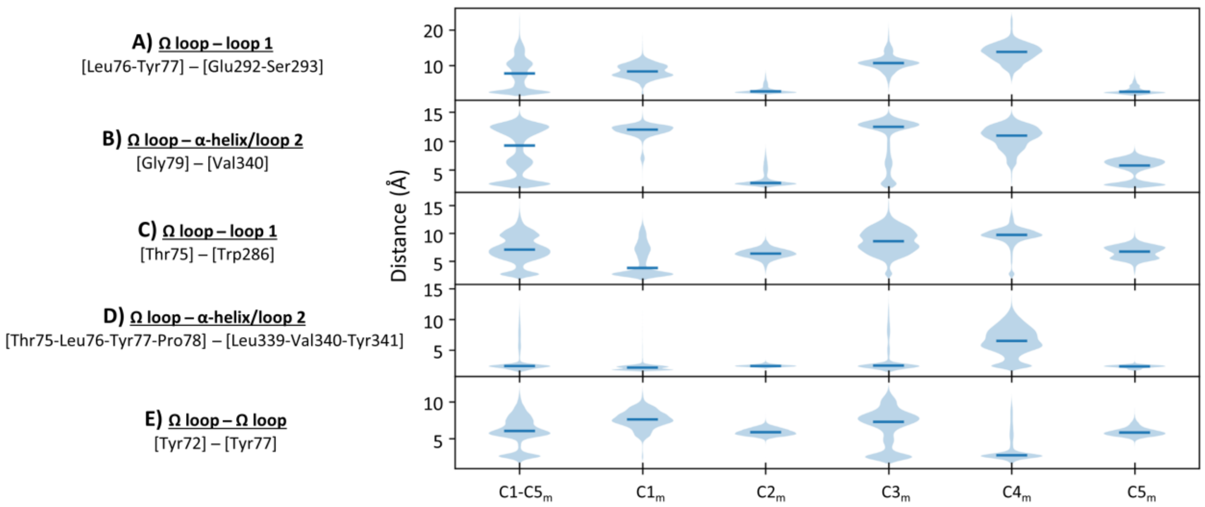

Figure S7. The pairwise minimum distances were calculated as the minimum distances between all atoms of selected residues in the loops, as extension of Figure S6. **A)** The Ω loop in relation to loop 1. **B)** The Ω loop in relation to the α-helix/loop 2. **C)** The Ω loop in relation to loop 1. **D)** The Ω loop in relation to α-helix/loop 2. **E)** The rearrangement of the Ω loop. The distances were calculated for the conformations of the original MD trajectory (50-500 ns). The dark blue line represents the mean value of the minimum distance throughout the MD simulation, and the width of the light blue shape represents the fraction of frames at each distance.

Gorge radius calculations

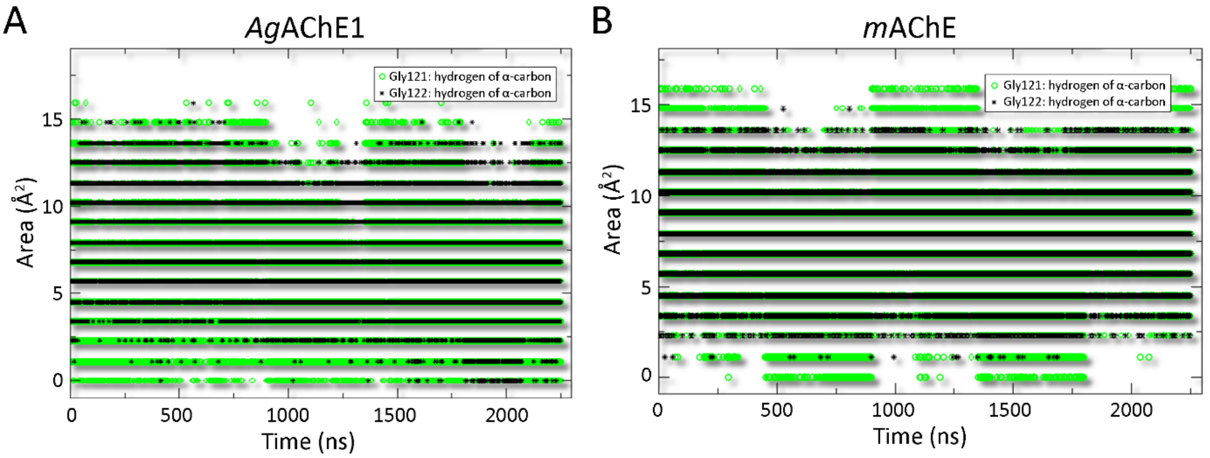

Figure S8. Exposed area of the hydrogen atom on the α-carbon of Gly121 or Gly122, calculated over the MD simulations of **A)** *Ag*AChE1 and **B)** *m*AChE. The hydrogen atom of Gly122 remained exposed throughout the simulations, thus it was selected as the seed coordinate for the radius calculations.

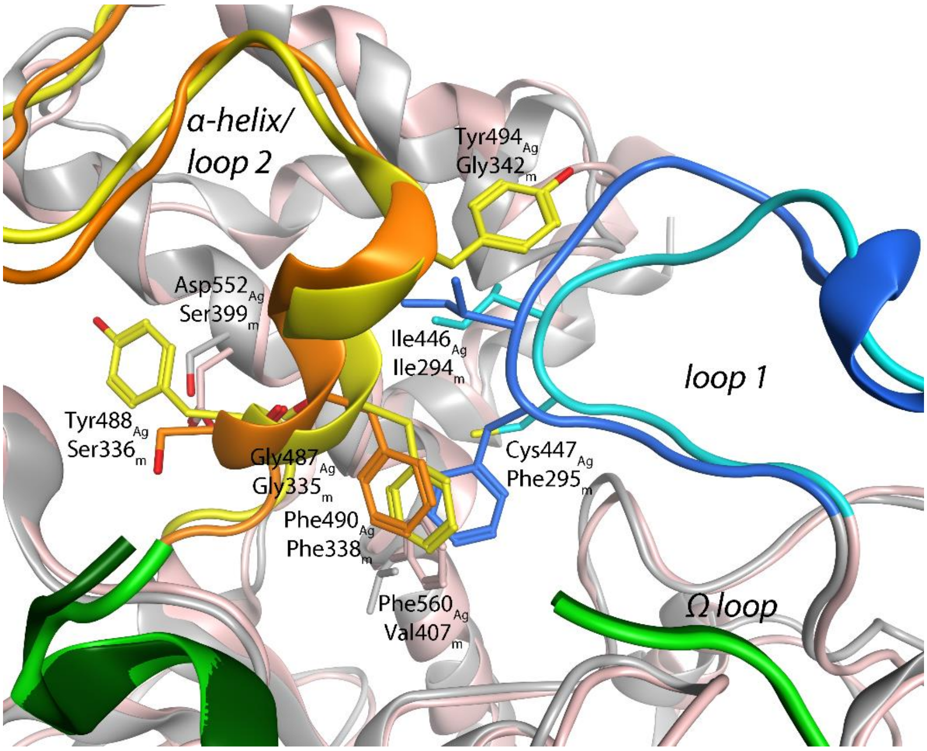

Figure S9. Structures displaying interactions formed to the α-helix/loop 2 of *Ag*AChE1 and *m*AChE, monitored in Figures S10, S11, and S12. The interactions result in a slightly different positioning of the α-helix. The figures are constructed based on the X-ray crystal structure of *Ag*AChE1 (PDB: 5X61) and *m*AChE (PDB: 1J06). *Ag*AChE1 and *m*AChE are displayed with pink or grey ribbons, respectively, with the Ω loop in green/dark green, loop 1 in cyan/blue, and α-helix/loop 2 in yellow/orange. Carbons are colored according to the ribbon, oxygen in red, nitrogen in blue, and sulfur in yellow. Part of the Ω loop has been removed to better display the residues of interest.

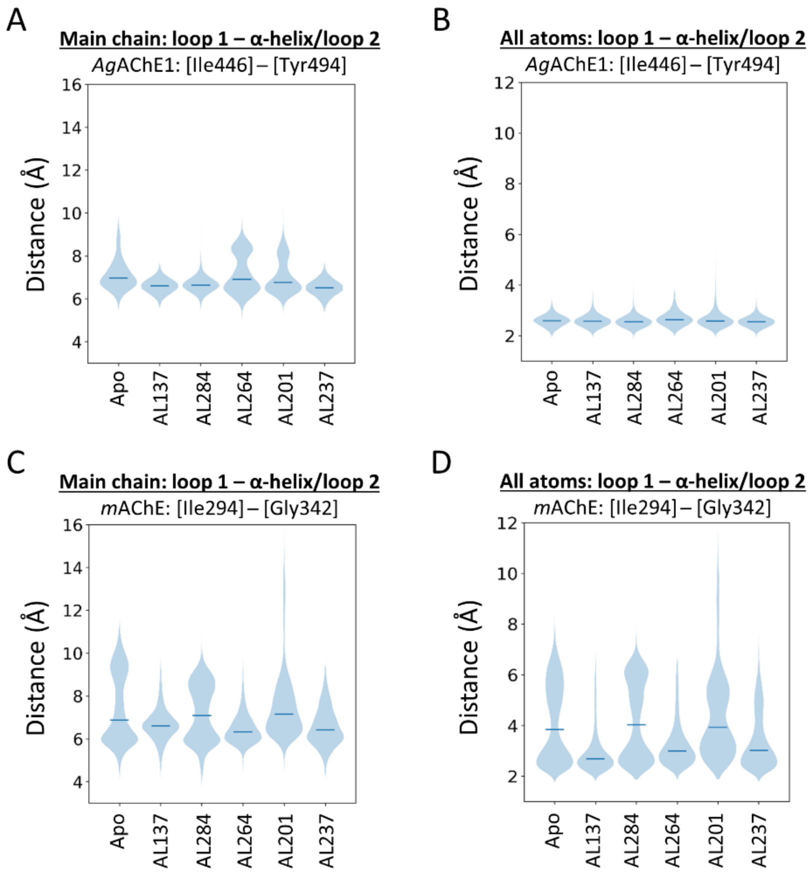

Figure S10. Pairwise minimum distances between selected amino acid residues in the loop 1 and α-helix/loop 2. For *Ag*AChE1, the minimum distance between the main chain atoms (**A**) and all atoms (**B**) of Ile446 (loop 1) and Tyr494 (α-helix/loop 2) are shorter than the corresponding distances for *m*AChE (Ile294-Gly342; **C-D**), which are also more varying. The distances were calculated for the conformations of the original MD trajectory (50-500 ns). The dark blue line represents the mean value of the minimum distance throughout the MD simulation, and the width of the light blue shape represents the fraction of frames at each distance.

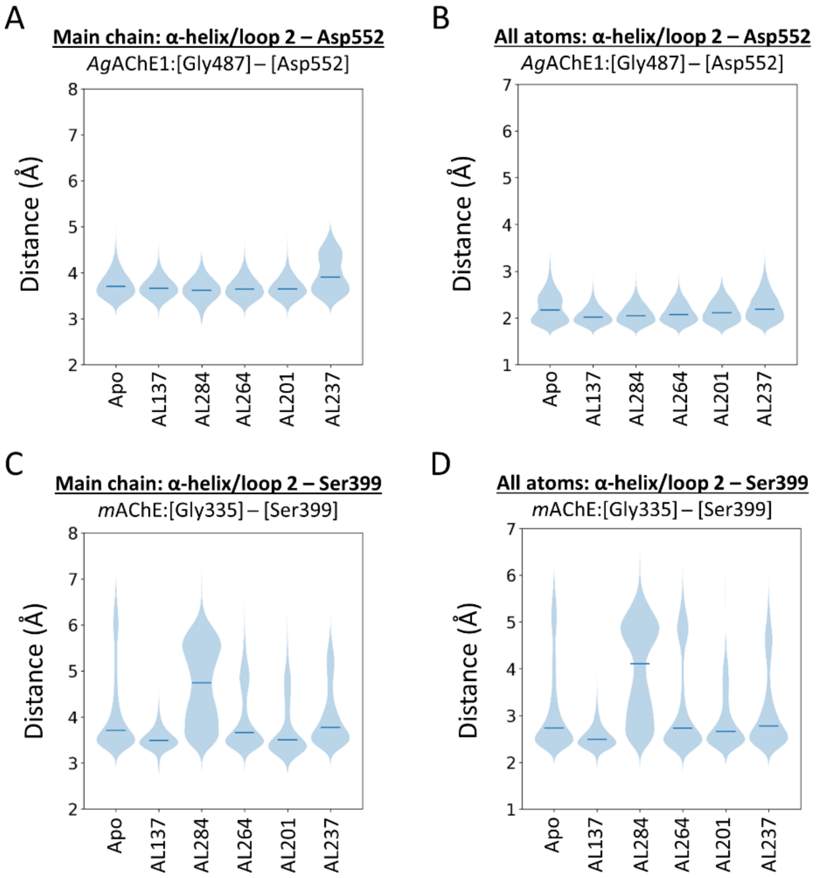

Figure S11. Pairwise minimum distances between selected amino acid residues in the α-helix/loop 2 and an adjacent α-helix at S3. For *Ag*AChE1, the minimum distance between the main chain atoms (**A**) and all atoms (**B**) of Gly487 (α-helix/loop 2) and Asp552 (adjacent α-helix) are shorter than the corresponding distances for *m*AChE (Gly335-Ser399; **C-D**), which are also more varying. The distances were calculated for the conformations of the original MD trajectory (50-500 ns). The dark blue line represents the mean value of the minimum distance throughout the MD simulation, and the width of the light blue shape represents the fraction of frames at each distance.

.

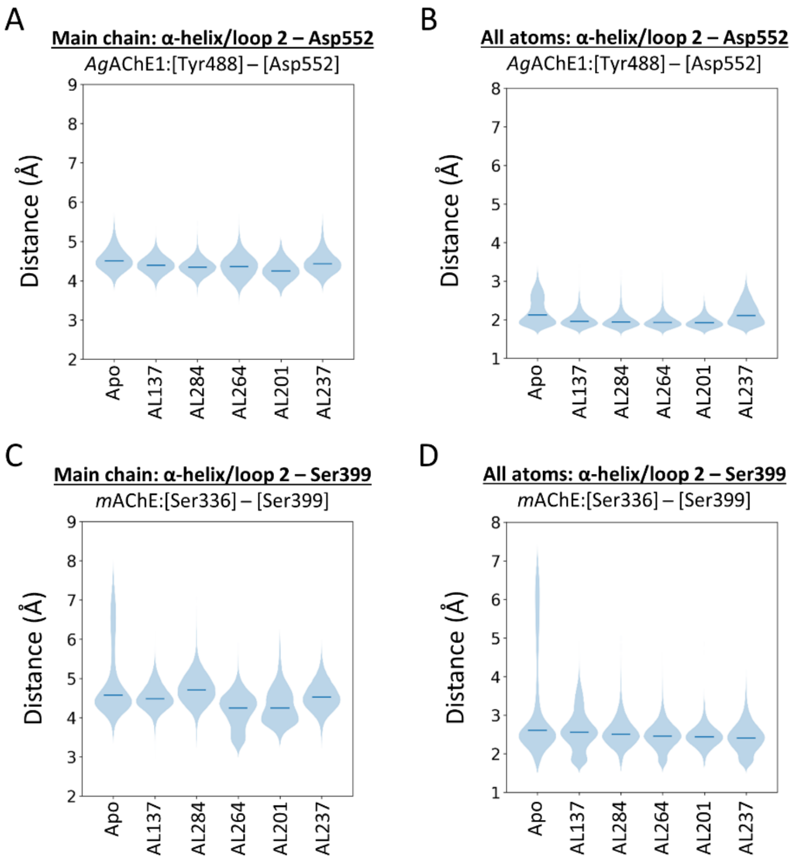

Figure S12. Pairwise minimum distances between selected amino acid residues in the α-helix/loop 2 and an adjacent α-helix at S3. For *Ag*AChE1, the minimum distance between the main chain atoms (**A**) and all atoms (**B**) of Tyr488 (α-helix/loop 2) and Asp552 (adjacent α-helix) are shorter than the corresponding distances for *m*AChE (Ser336-Ser399; **C-D**), which are also more varying. The distances were calculated for the conformations of the original MD trajectory (50-500 ns). The dark blue line represents the mean value of the minimum distance throughout the MD simulation, and the width of the light blue shape represents the fraction of frames at each distance.

Crystal structure of *m*AChE•AL237

Table S6. Data collection and refinement statistics of *m*AChE•AL237 (pdb entry code 8ORC).[a]

Resolution range (Å)	49.23 - 2.1 (2.175 - 2.1)
Space group	P 21 21 21
Unit cell (Å)	79.606 112.113 226.605 90 90 90
Total reflections	865025 (72173)
Unique reflections	118925 (11746)
Multiplicity	7.3 (6.1)
Completeness (%)	99.81 (99.71)
Mean I/sigma (I)	18.58 (2.78)
Wilson B-factor	34.21
R-merge	0.08429 (0.8978)
R-meas	0.09086 (0.9823)
R-pim	0.03368 (0.3954)
CC1/2	0.999 (0.87)
CC*	1 (0.965)
Reflections used in refinement	118753 (11713)
Reflections used for R-free	2379 (250)
R-work	0.1860 (0.2800)
R-free	0.2065 (0.2930)
CC (work)	0.960 (0.908)
CC (free)	0.937 (0.890)
Number of non-hydrogen atoms	9056
macromolecules	8380
ligands	333
solvent	537
Protein residues	1070

RMS (bonds)	0.005
RMS (angles)	0.71
Ramachandran favored (%)	95.95
Ramachandran allowed (%)	3.48
Ramachandran outliers (%)	0.56
Rotamer outliers (%)	0.23
Clashscore	4.68
Average B-factor	51.12
macromolecules	50.45
ligands	69.00
solvent	56.86
Number of TLS groups	13

[a]Statistics for the highest-resolution shell are shown in parentheses.

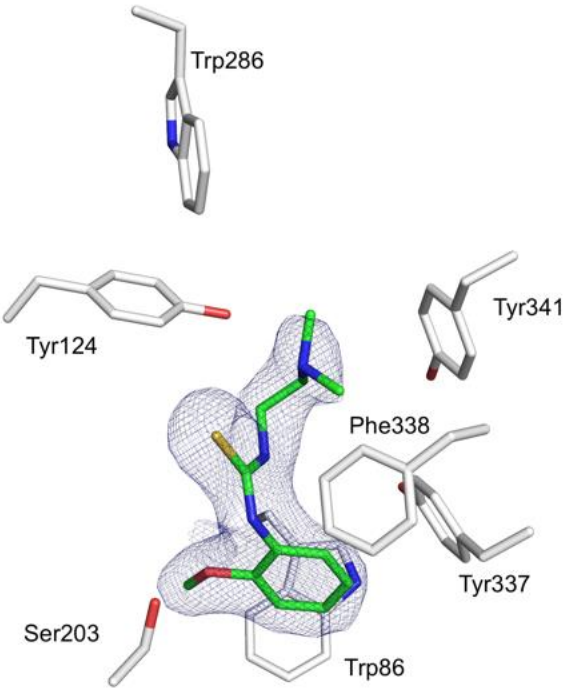

Figure S13. The Fo-Fc simulated annealing omit electron density map at a contour level of 3.5 defining the binding pose of AL237 binding to *m*AChE. The AL237 molecule is shown in green, *m*AChE in grey and the electron density map in blue.

LigPlot diagrams of *m*AChE•inhibitor complexes

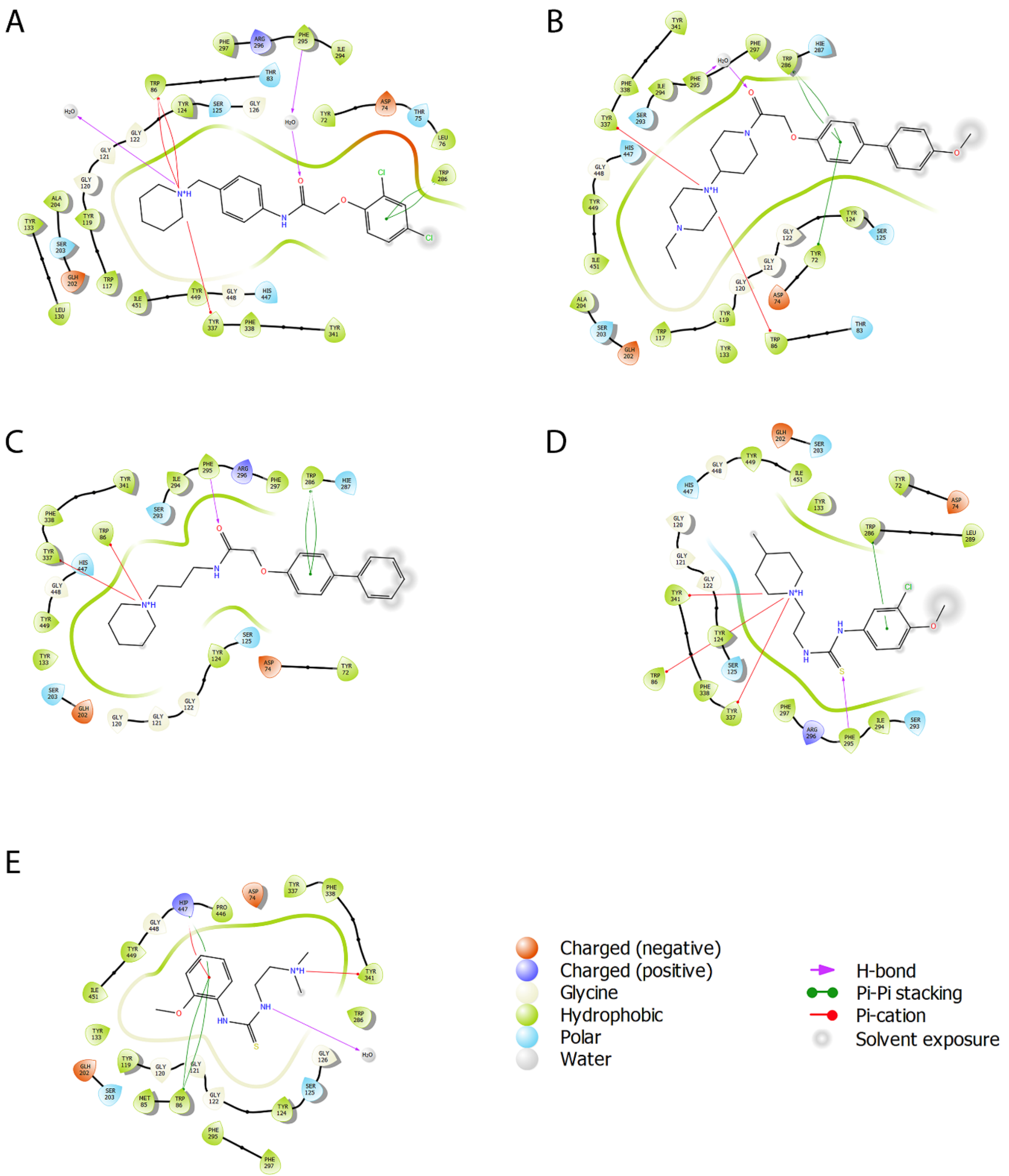

Figure S14. LigPlot diagrams for *m*AChE1•AL137 (**A**), *m*AChE•AL284 (**B**), *m*AChE•AL264 (**C**), *m*AChE•AL201 (**D**), and *m*AChE•AL237 (**E**). The diagrams are based on the crystal structures, except for AL201 which was modelled in silico in the binding site of *m*AChE, taking the experimentally determined electron density map into account, and made using Schrödinger: Maestro (Version 13.1.141, Release 2022-1).

RMSD-plots of MD simulations of inhibited *Ag*AChE1 and *m*AChE

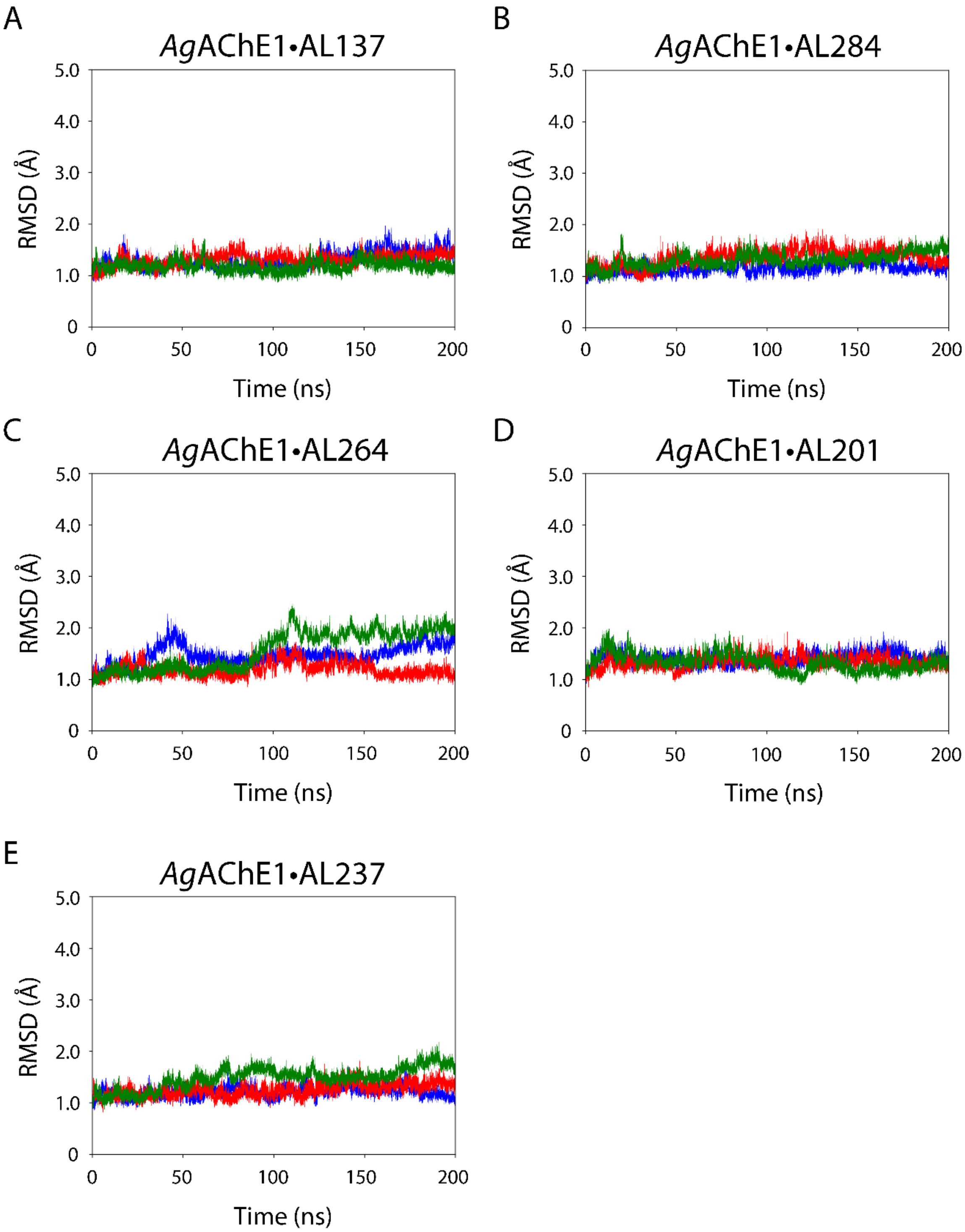

Figure S15. Root mean square deviation (RMSD) values of *Ag*AChE1 protein backbone atoms vs simulation time for the three (**A-E**) separate MD simulations, calculated against the equilibrated structure for **A**) *Ag*AChE1•AL137, **B**) *Ag*AChE1• AL284, **C**) *Ag*AChE1•AL264, **D**) *Ag*AChE1•AL201, and **E**) *Ag*AChE1•AL237. The simulations were considered to be converged after 50 ns, thus the last 150 ns of the trajectories were used in further analysis.

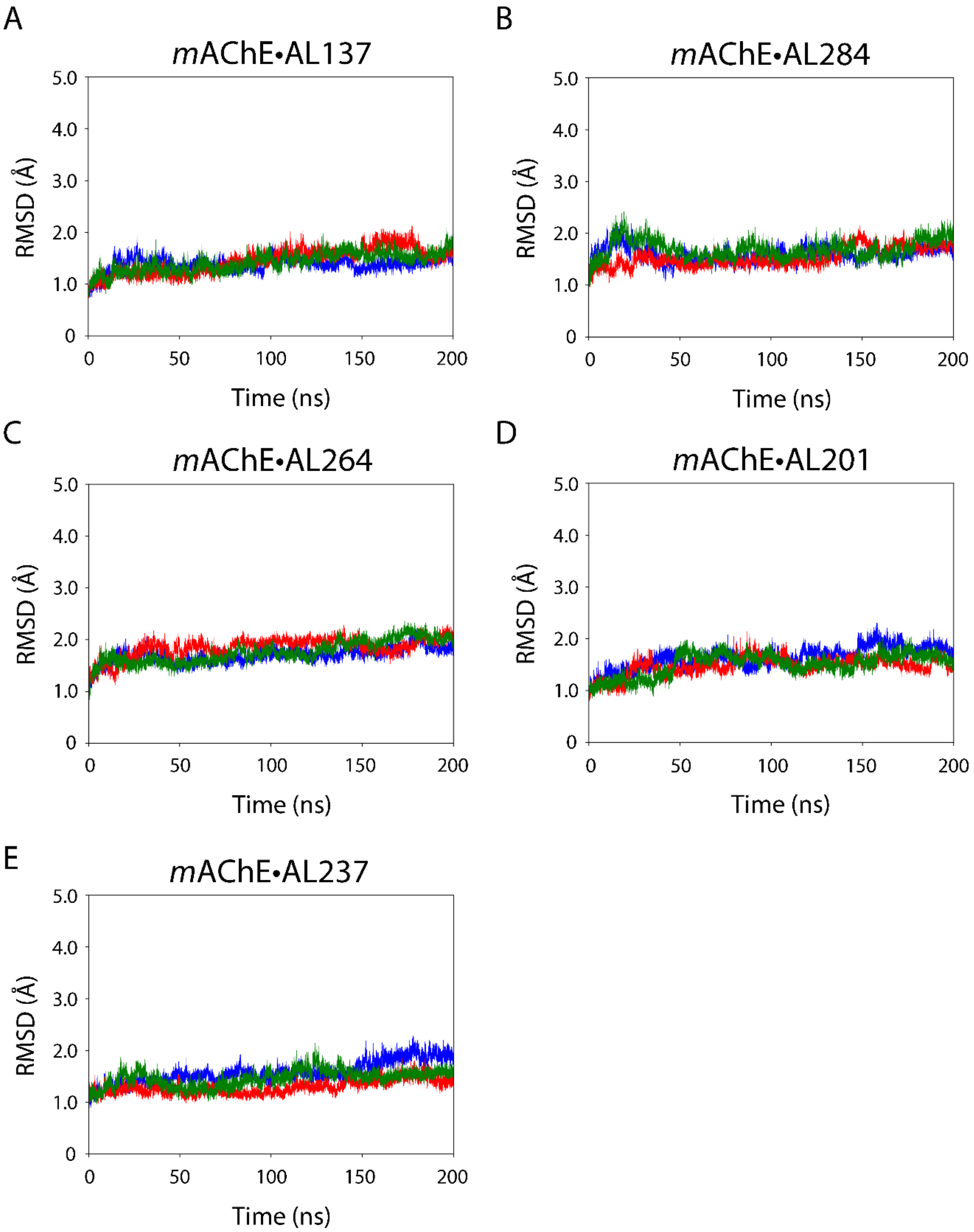

Figure S16. Root mean square deviation (RMSD) values of *m*AChE protein backbone atoms vs simulation time for three (**A-E**) separate simulations, calculated against the equilibrated structure for **A**) *m*AChE•AL137, **B**) *m*AChE•AL284, **C**) *m*AChE•AL264, **D**) *m*AChE•AL201, and **E**) *m*AChE•AL237. The simulations are considered to be converged after 50 ns, thus the last 450 or 150 ns of the trajectories were used in further analysis.

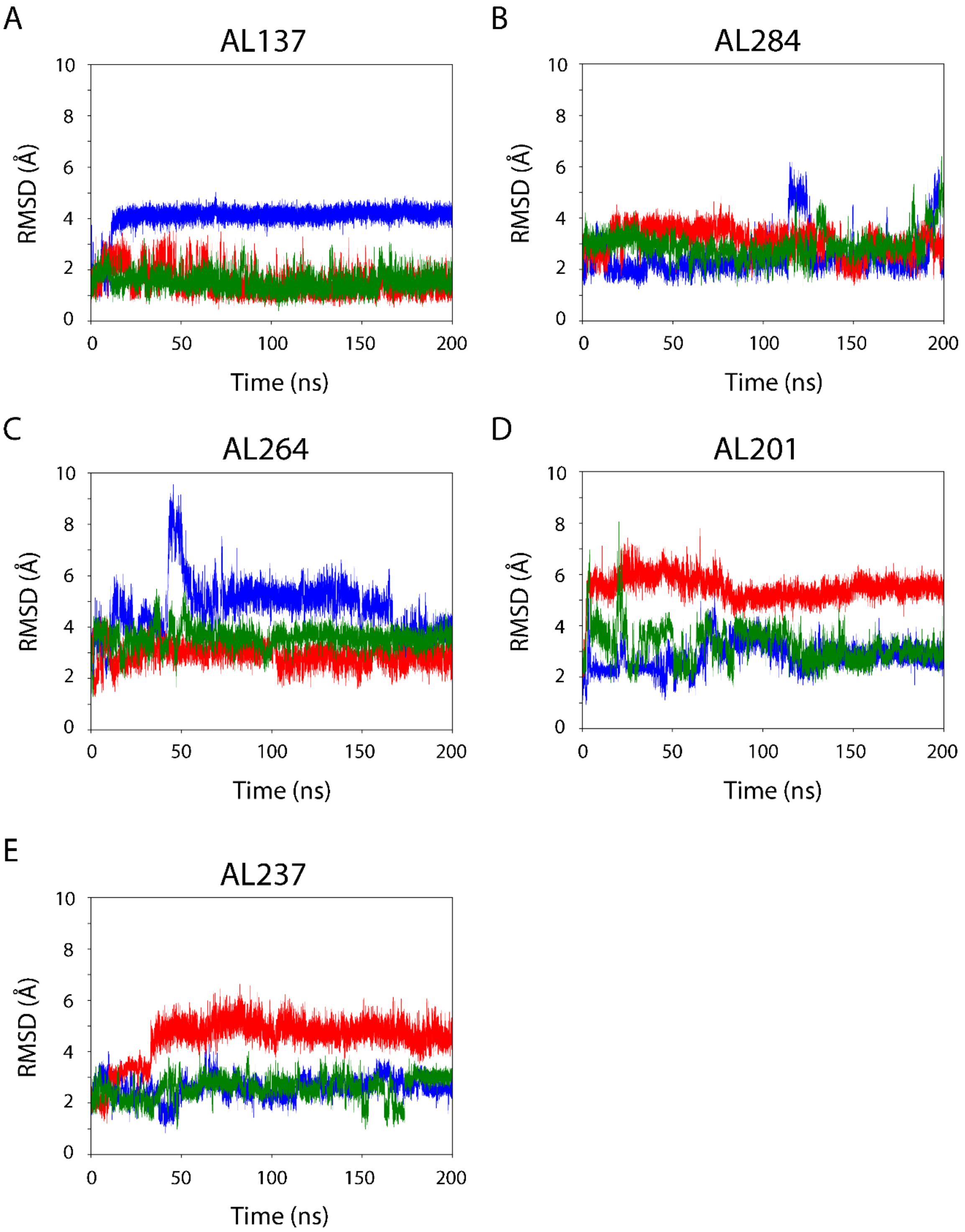

Figure S17. Root mean square deviation (RMSD) values of the inhibitors' heavy atoms vs simulation time for the three separate simulations, calculated against the equilibrated structure for **A**) *Ag*AChE1•AL137, **B**) *Ag*AChE1•AL284, **C**) *Ag*AChE1•AL264, **D**) *Ag*AChE1•AL201, and **E**) *Ag*AChE1•AL237.

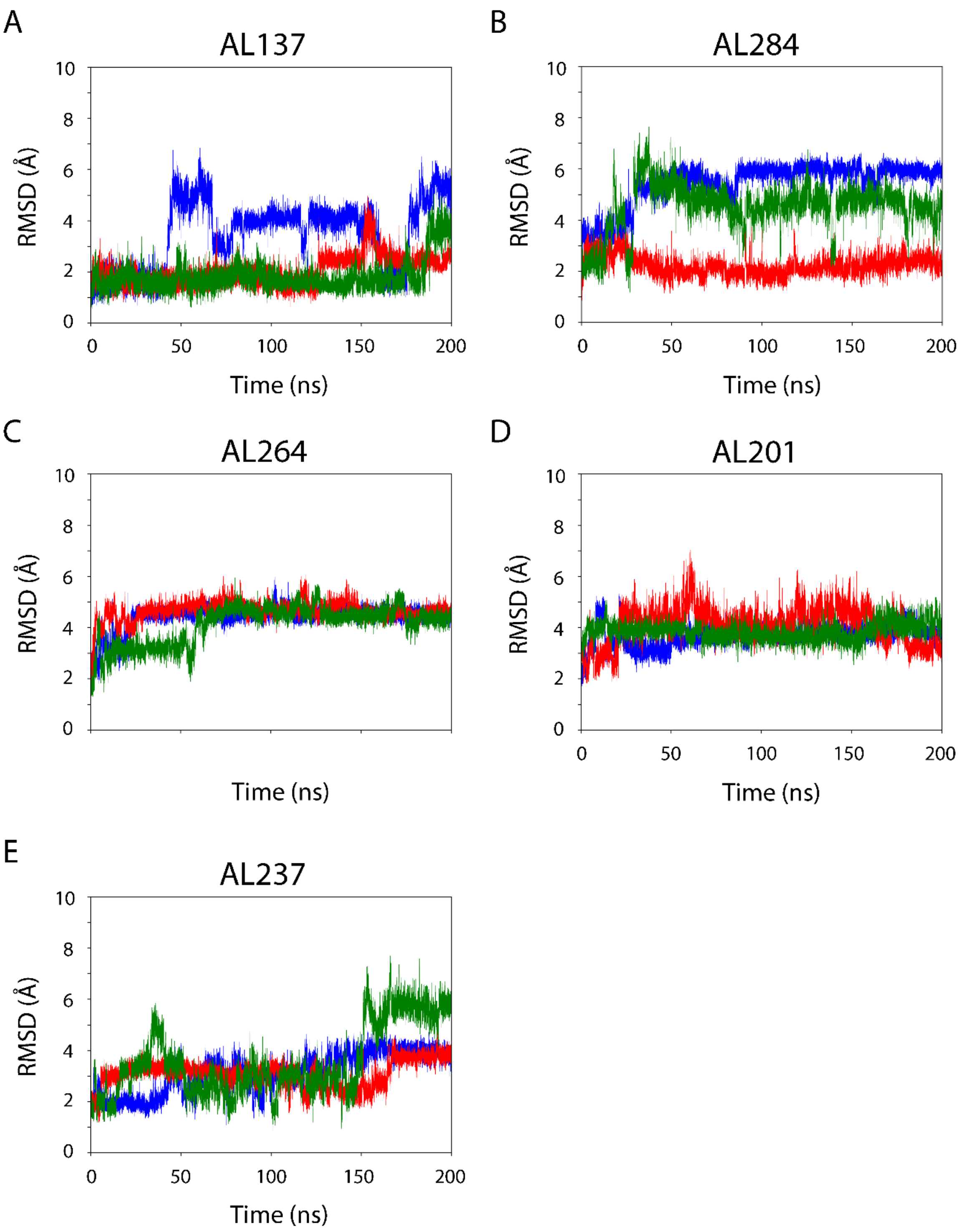

Figure S18. Root mean square deviation (RMSD) values of the inhibitors' heavy atoms vs simulation time for the three separate simulations, calculated against the equilibrated structure for **A**) *m*AChE•AL137, **B**) *m*AChE•AL284, **C**) *m*AChE•AL264, **D**) *m*AChE•AL201, and **E**) *m*AChE•AL237.

Investigation of largest collective motions for AChE•inhibitor complexes

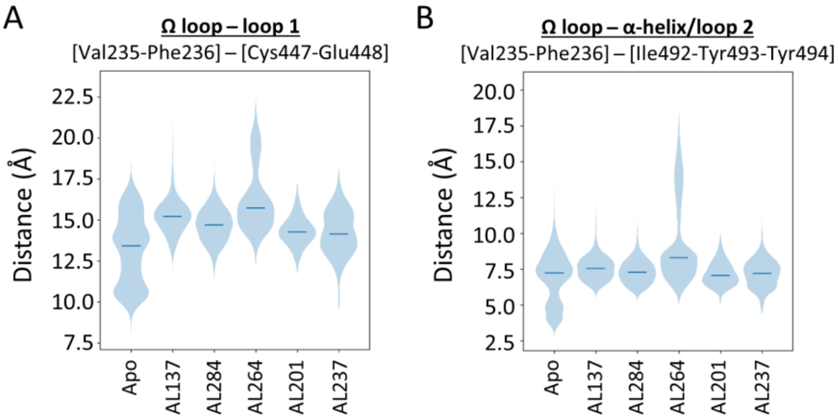

Figure S19. Pairwise minimum distances between main chain atoms of selected amino acid residues of apo *Ag*AChE1 and *Ag*AChE1•inhibitor complexes in the Ω loop and loop 1 (**A**) and the α-helix/loop 2 (**B**), respectively. The distances are longer for the *Ag*AChE1•AL264 complex compared to the others. The distances were calculated for the conformations of the original MD trajectory (50-500 ns (apo) and 50-150 ns (inhibitor complex)). The dark blue line represents the mean value of the minimum distance throughout the MD simulation, and the width of the light blue shape represents the fraction of frames at each distance.

The effect of inhibitors on local enzyme dynamics

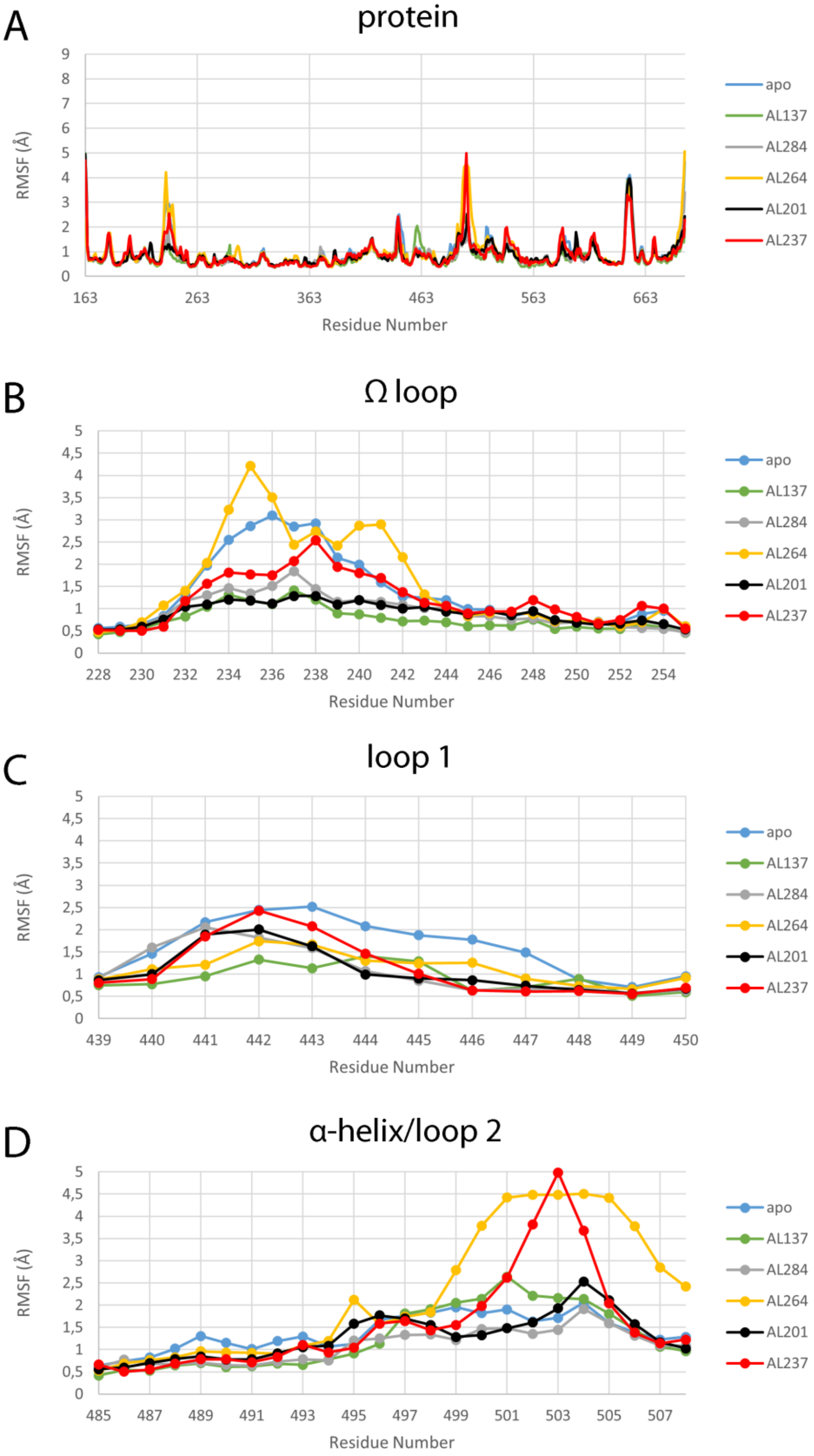

Figure S20. RMSF values calculated for the backbone residues of the apo form of *Ag*AChE1 and *Ag*AChE1•inhibitor complexes (**A**), zooming in on the three loop regions Ω loop (**B**), loop 1 (**C**), and α-helix/loop 2 (**D**). The RMSF values show that the fluctuations of the Ω loop and the α-helix/loop 2 were affected by the inhibitors.

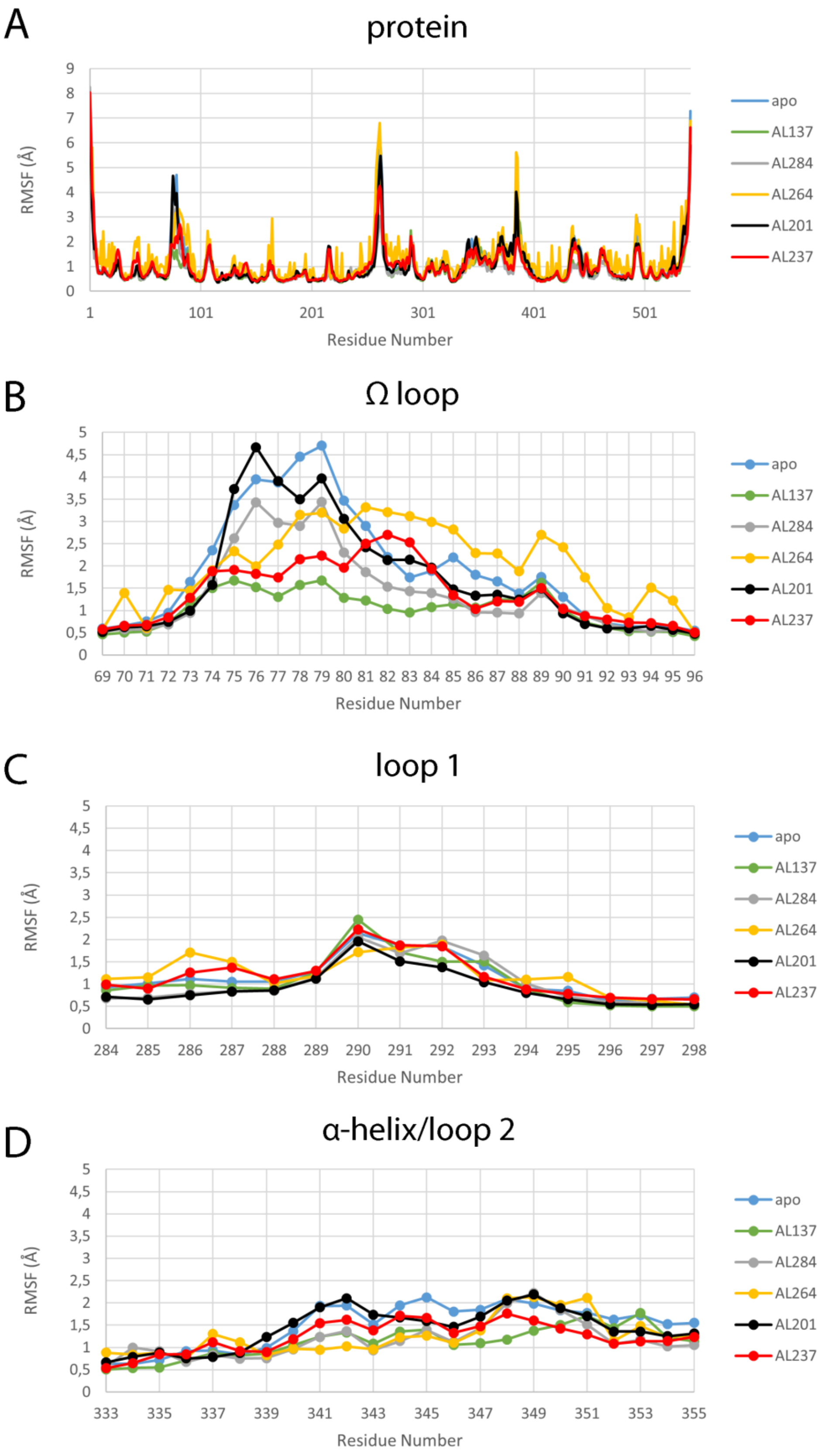

Figure S21. RMSF values calculated for the backbone residues of the apo form of *m*AChE and *m*AChE•inhibitor complexes (**A**), zooming in on the three loop regions Ω loop (**B**), loop 1 (**C**), and α-helix/loop 2 (**D**). The RMSF values show that the fluctuations of the Ω loop is affected by the inhibitors.

Waters in the active site gorges of *Ag*AChE1 and *m*AChE

Table S7. Crystal structures of *m*AChE included in the water analysis

No	PDB code	Resolution
1	5DTI	2.003 Å
2	2HA2	2.05 Å
3	4B82	2.1 Å
4	4B85	2.1 Å
5	5EIE	2.1 Å
6[a]	8ORC	2.1 Å
7	2HA5	2.15 Å
8	2WHQ	2.15 Å
9	1N5M	2.2 Å
10	2GYU	2.2 Å
11	2HA0	2.2 Å
12	2WHP	2.2 Å
13	1N5R	2.25 Å
14	2HA3	2.25 Å
15	2HA6	2.25 Å
16	4ARB	2.25 Å
17	4B7Z	2.3 Å
18[b]	5FOQ	2.3 Å
19	7R02	2.3 Å
20	7R2F	2.3 Å

[a]The complex *m*AChE•AL237. [b]The complex *m*AChE•AL137.

Table S8. Crystal structures of *h*AChE included in the water analysis

No	PDB code	Resolution
1	4M0E	2 Å
2	6NTO	2.052 Å
3	6O69	2.081 Å
4	5HF5	2.152 Å
5	6O5V	2.152 Å
6	4EY4	2.156 Å
7	6WVO	2.19 Å
8	5HF9	2.2 Å
9	5HFA	2.201 Å
10	6CQZ	2.216 Å
11	6NTL	2.25 Å
12	6U37	2.25 Å
13	6WUZ	2.253 Å
14	6CQT	2.273 Å
15	6CQW	2.278 Å

16	6WVQ	2.289 Å
17	5HF6	2.3 Å
18	6O4X	2.3 Å
19	4EY5	2.3012 Å
20	4M0F	2.304 Å

Table S9. Selected conserved crystal waters and their corresponding amino acid residue resulting in putative hydrogen bond

*m*ACHE			*h*AChE		H-bond		
Waters (PDB: 4B82)[a]	Waters (PDB: 5FOQ)[b]	Frequence	Waters (PDB: 6O5V)[a]	Frequence	aa residue[c]	Heavy atom[d]	ID
HOH2189	HOH2056	20/20	HOH864	20/20	Gly120	N	G1
HOH2190	HOH2057	20/20	HOH746	19/20	Gly120	O	G2
HOH2199	HOH2061	19/20	HOH803	19/20	Ser125	O[e]	G3
HOH2122	-[f]	13/20	HOH738	14/20	Tyr124	O[e]	G4
HOH2139	HOH2044	20/20	HOH1014	19/20	Trp86	O	O1
HOH2127	HOH2036	19/20	HOH876	20/20	Thr83	O	O2
HOH2115	-[f]	19/20	HOH771	20/20	Tyr72	O	O3
HOH2117	-[f]	14/20	-[f]	14/20	Thr75	N	O4
HOH2194	HOH2059	19/20	HOH822	20/20	Gly122	O	L1
HOH2340	-[f]	17/20	HOH710	20/20	Phe299	N	L2
HOH2356	HOH2120	15/20	HOH934	17/20	Arg296	O	L3
					Arg296	N	L4
HOH2359	HOH2121	14/20[g]	-[f]	7/20[h]	Phe295	N	L5

[a]Complexes with a resolution ≤ 2.1 Å and a non-covalent inhibitor were chosen to represent the conserved waters in *m*AChE and *h*AChE (PDB: 4B82 and 6O5V, respectively). [b]The complex *m*AChE•AL137. [c]Amino acid residues are numbered according to *m*AChE. [d]Atoms in the backbone if not noted otherwise. [e]Atoms in the side chains. [f]Not present in the crystal structure. [g]In five additional crystal structures, the water position was occupied by another atom (ligand or ion). [h]In eleven additional crystal structures, the water position was occupied by another atom (ligand, ion or loop movement).

.

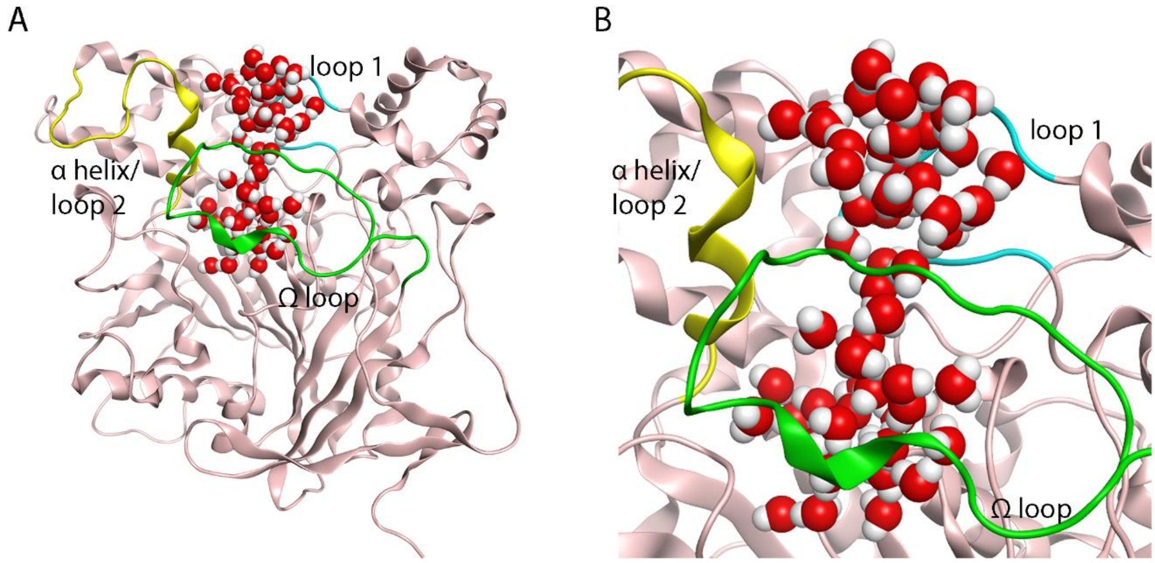

Figure S22. Waters occupying the active site gorge of *Ag*AChE1 based on the equilibrated structure that was submitted to the MD simulations (frame 0). The overview figure **(A)** and the close up **(B)** show that the deep gorge is filled with waters, where the active site and the peripheral sites are connected via a narrow waist populated with a single water.

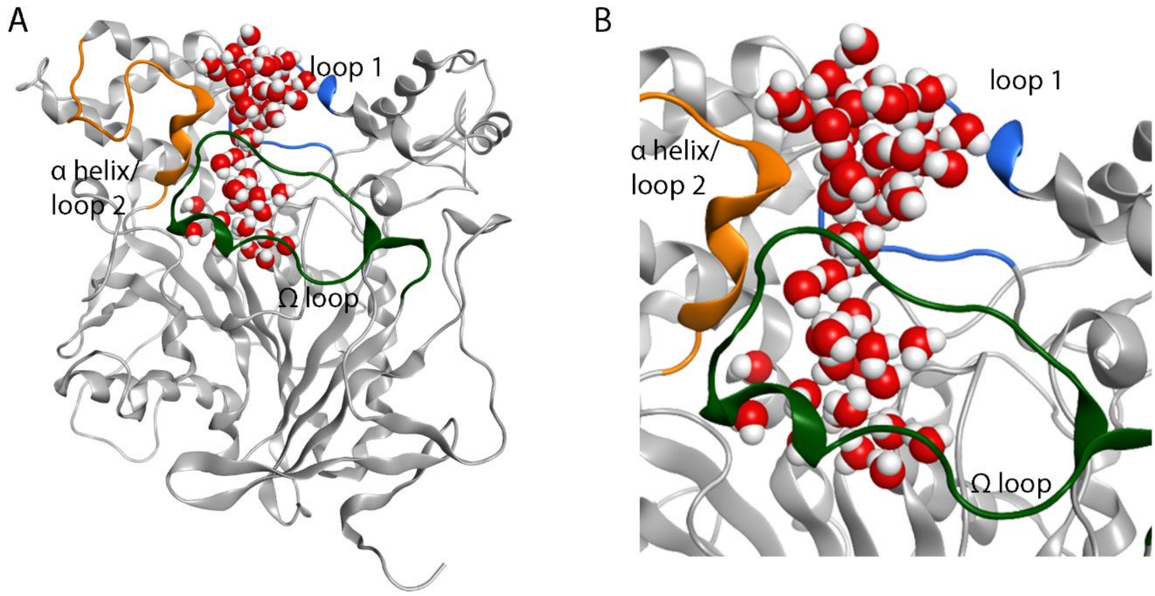

Figure S23. Waters occupying the active site gorge of *m*AChE based on the equilibrated structure that was submitted to the MD simulations (frame 0). The overview figure **(A)** and the close up **(B)** show that the deep gorge is filled with waters. where the active site and the peripheral sites are connected via a narrow waist populated with a single water.

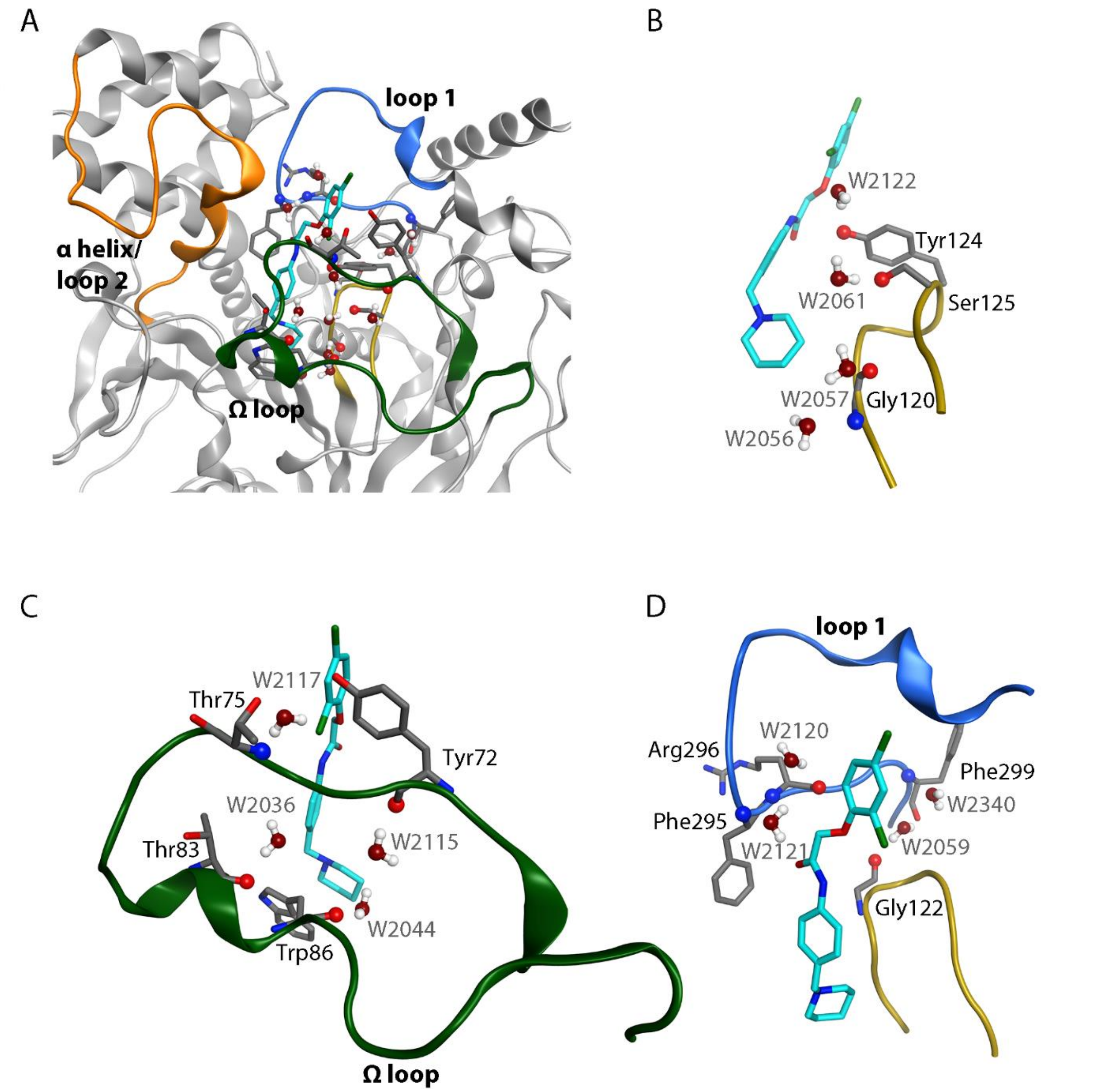

Figure S24. The selected waters and amino acid residue atoms listed in Table S9 based on *m*AChE•AL137 (PDB: 5FOQ). **A)** Overview of all waters and residues included in relation to AL137 and the three loops at the entrance of the gorge. **B)** Waters reaching from the bottom of the gorge up to the narrow gorge waist (G1-G4). **C)** Waters interacting with the Ω loop (O1-O4). **D)** Waters interacting with Gly122 and the loop 1 (L1-L4). The four conserved waters that are missing in PDB: 5FOQ have been added by coordinates and water numbers from PDB: 4B82.

Table S10. Water occupancy at hydrogen bonding distances (< 3.5 Å) to amino acid residue atoms of *Ag*AChE1 calculated as averaged number of water molecules over the MD trajectories[a]

ID	Atom	*Ag*AChE1 structure[b]					
		Apo	AL137	AL284	AL264	AL201	AL237
G1	$Gly278_{Ag}/Gly120_{m}$: N	0.67	0.86	1.20	0.93	0.88	0.86
G2	$Gly278_{Ag}/Gly120_{m}$: O	0.91	**1.14**	**1.25**	0.92	**1.03**	0.64
G3	$Ser283_{Ag}/Ser125_{m}$: O(H)	1.41	1.44	1.37	1.34	1.38	1.02
G4	$Tyr282_{Ag}/Tyr124_{m}$: O(H)	2.75	**1.96**	**1.96**	1.32	**1.91**	1.63
O1	$Trp245_{Ag}/Trp86_{m}$: O	1.55	1.86	1.85	1.78	1.77	1.52
O2	$Thr242_{Ag}/Thr83_{m}$: O	0.72	1.19	1.22	1.42	1.05	0.99
O3	$Ile231_{Ag}/Tyr72_{m}$: O	1.06	1.15	1.21	1.17	1.24	1.15
O4	$Thr234_{Ag}/Thr75_{m}$: N	1.05	1.17	0.95	0.69	0.99	0.92
L1	$Gly280_{Ag}/Gly122_{m}$: O	0.84	0.53	0.75	0.79	0.86	0.73
L2	$Phe451_{Ag}/Phe299_{m}$: N	0.99	0.96	0.94	1.03	0.94	1.15
L3	$Glu448_{Ag}/Arg296_{m}$: O	1.72	2.50	1.61	1.61	1.98	1.67
L4	$Glu448_{Ag}/Arg296_{m}$: N	0.34	0.33	0.35	0.32	0.31	0.37
L5	$Cys447_{Ag}/Phe295_{m}$: N	0.75	0.64	1.05	0.69	0.71	1.02

[a]The first 50 ns of the simulations were excluded in the analysis. [b]Water occupancy is color coded in range of 0.30-2.75, going from dark red via white to dark blue.

Table S11. Water occupancy at hydrogen bonding distances (< 3.5 Å) to amino acid residue atoms of *m*AChE calculated as averaged number of water molecules over the MD trajectories[a]

ID	Atom	*m*AChE structure					
		Apo	AL137	AL284	AL264	AL201	AL237
G1	$Gly278_{Ag}/Gly120_{m}$: N	0.59	0.70	0.88	0.65	0.72	0.94
G2	$Gly278_{Ag}/Gly120_{m}$: O	1.64	1.57	1.20	1.60	0.80	1.58
G3	$Ser283_{Ag}/Ser125_{m}$: O(H)	0.97	1.26	1.07	0.89	0.47	0.92
G4	$Tyr282_{Ag}/Tyr124_{m}$: O(H)	2.62	1.95	1.03	1.09	1.22	1.73
O1	$Trp245_{Ag}/Trp86_{m}$: O	0.99	1.54	1.53	1.45	0.99	1.70
O2	$Thr242_{Ag}/Thr83_{m}$: O	0.52	0.77	0.91	1.11	0.50	0.85
O3	$Ile231_{Ag}/Tyr72_{m}$: O	1.05	1.05	1.16	0.77	1.10	1.15
O4	$Thr234_{Ag}/Thr75_{m}$: N	0.59	0.61	0.88	0.93	0.61	0.92
L1	$Gly280_{Ag}/Gly122_{m}$: O	0.81	0.83	0.96	0.97	0.86	0.73
L2	$Phe451_{Ag}/Phe299_{m}$: N	1.07	1.28	1.35	1.22	1.41	1.15
L3	$Glu448_{Ag}/Arg296_{m}$: O	1.80	1.43	1.96	1.10	1.12	1.67
L4	$Glu448_{Ag}/Arg296_{m}$: N	0.57	0.66	0.75	0.84	0.57	0.37
L5	$Cys447_{Ag}/Phe295_{m}$: N	0.92	1.09	1.30	1.49	0.97	1.02

[a]The first 50 ns of the simulations were excluded in the analysis. [b]Water occupancy is color coded in range of 0.30-2.75, going from dark red via white to dark blue.

Table S12. Water occupancy at profound hydrogen bonding distances (< 3.0 Å) to amino acid residue atoms of *Ag*AChE1 calculated as averaged number of water molecules over the MD trajectories[a]

ID	Atom	*Ag*AChE1 structure					
		Apo	AL137	AL284	AL264	AL201	AL237
G1	$Gly278_{Ag}/Gly120_{m}$: N	0.33	0.36	0.53	0.55	0.51	0.49
G2	$Gly278_{Ag}/Gly120_{m}$: O	0.49	0.91	0.71	0.58	0.58	0.47
G3	$Ser283_{Ag}/Ser125_{m}$: O(H)	0.84	0.90	0.83	0.81	0.86	0.64
G4	$Tyr282_{Ag}/Tyr124_{m}$: O(H)	1.42	1.03	1.16	0.88	0.99	0.95
O1	$Trp245_{Ag}/Trp86_{m}$: O	1.04	1.17	1.15	1.12	1.14	1.08
O2	$Thr242_{Ag}/Thr83_{m}$: O	0.49	0.66	0.66	0.80	0.74	0.66
O3	$Ile231_{Ag}/Tyr72_{m}$: O	0.87	0.99	0.94	0.91	1.00	0.89
O4	$Thr234_{Ag}/Thr75_{m}$: N	0.26	0.34	0.33	0.23	0.26	0.23
L1	$Gly280_{Ag}/Gly122_{m}$: O	0.56	0.29	0.55	0.56	0.50	0.48
L2	$Phe451_{Ag}/Phe299_{m}$: N	0.33	0.34	0.29	0.36	0.19	0.39
L3	$Glu448_{Ag}/Arg296_{m}$: O	1.40	1.79	1.35	1.33	1.56	1.42
L4	$Glu448_{Ag}/Arg296_{m}$: N	0.02	0.01	0.01	0.02	0.01	0.01
L5	$Cys447_{Ag}/Phe295_{m}$: N	0.31	0.30	0.60	0.30	0.36	0.49

[a]The first 50 ns of the simulations were excluded in the analysis. [b]Water occupancy is color coded in range of 0.00-1.80, going from dark red via white to dark blue.

Table S13. Water occupancy at profound hydrogen bonding distances (< 3.0 Å) to amino acid residue atoms of *m*AChE calculated as averaged number of water molecules over the MD trajectories[a]

ID	Atom	*m*AChE structure					
		Apo	AL137	AL284	AL264	AL201	AL237
G1	$Gly278_{Ag}/Gly120_{m}$: N	0.29	0.36	0.60	0.31	0.43	0.49
G2	$Gly278_{Ag}/Gly120_{m}$: O	1.01	0.91	0.81	0.89	0.59	1.05
G3	$Ser283_{Ag}/Ser125_{m}$: O(H)	0.54	0.83	0.80	0.57	0.27	0.54
G4	$Tyr282_{Ag}/Tyr124_{m}$: O(H)	1.41	1.04	0.72	0.52	0.76	0.93
O1	$Trp245_{Ag}/Trp86_{m}$: O	0.69	1.06	0.99	1.00	0.75	1.10
O2	$Thr242_{Ag}/Thr83_{m}$: O	0.32	0.48	0.48	0.71	0.29	0.53
O3	$Ile231_{Ag}/Tyr72_{m}$: O	0.80	0.86	0.92	0.61	0.88	0.75
O4	$Thr234_{Ag}/Thr75_{m}$: N	0.10	0.28	0.29	0.22	0.14	0.15
L1	$Gly280_{Ag}/Gly122_{m}$: O	0.57	0.60	0.67	0.68	0.54	0.46
L2	$Phe451_{Ag}/Phe299_{m}$: N	0.28	0.38	0.43	0.50	0.42	0.33
L3	$Glu448_{Ag}/Arg296_{m}$: O	1.21	1.02	1.26	0.91	0.85	1.22
L4	$Glu448_{Ag}/Arg296_{m}$: N	0.13	0.17	0.24	0.31	0.14	0.20
L5	$Cys447_{Ag}/Phe295_{m}$: N	0.34	0.39	0.50	0.57	0.40	0.47

[a]The first 50 ns of the simulations were excluded in the analysis. [b]Water occupancy is color coded in range of 0.00-1.80, going from dark red via white to dark blue.

Interactions between inhibitors and enzymes

Table S14. Clusters identified in the cluster analysis performed on protein-ligand interactions for each *Ag*AChE1•inhibitor and *m*AChE•inhibitor complex, using *gmx_clusterByFeatures*[1]

Inhibitor	*Ag*AChE1		*m*AChE	
	Number of clusters	Fraction of total frames in each cluster (%)	Number of clusters	Fraction of total frames in each cluster (%)
AL137	2	66/34	4	54/16/16/14
AL284	4	35/32/28/4	5	33/33/17/10/6
AL264	10	25/15/13/12/11/7/6/5/4/2	10	22/19/15/13/8/6/5/4/4/4
AL201	3	37/33/29	4	33/33/22/12
AL237	2	67/33	7	23/18/15/13/13/11/8

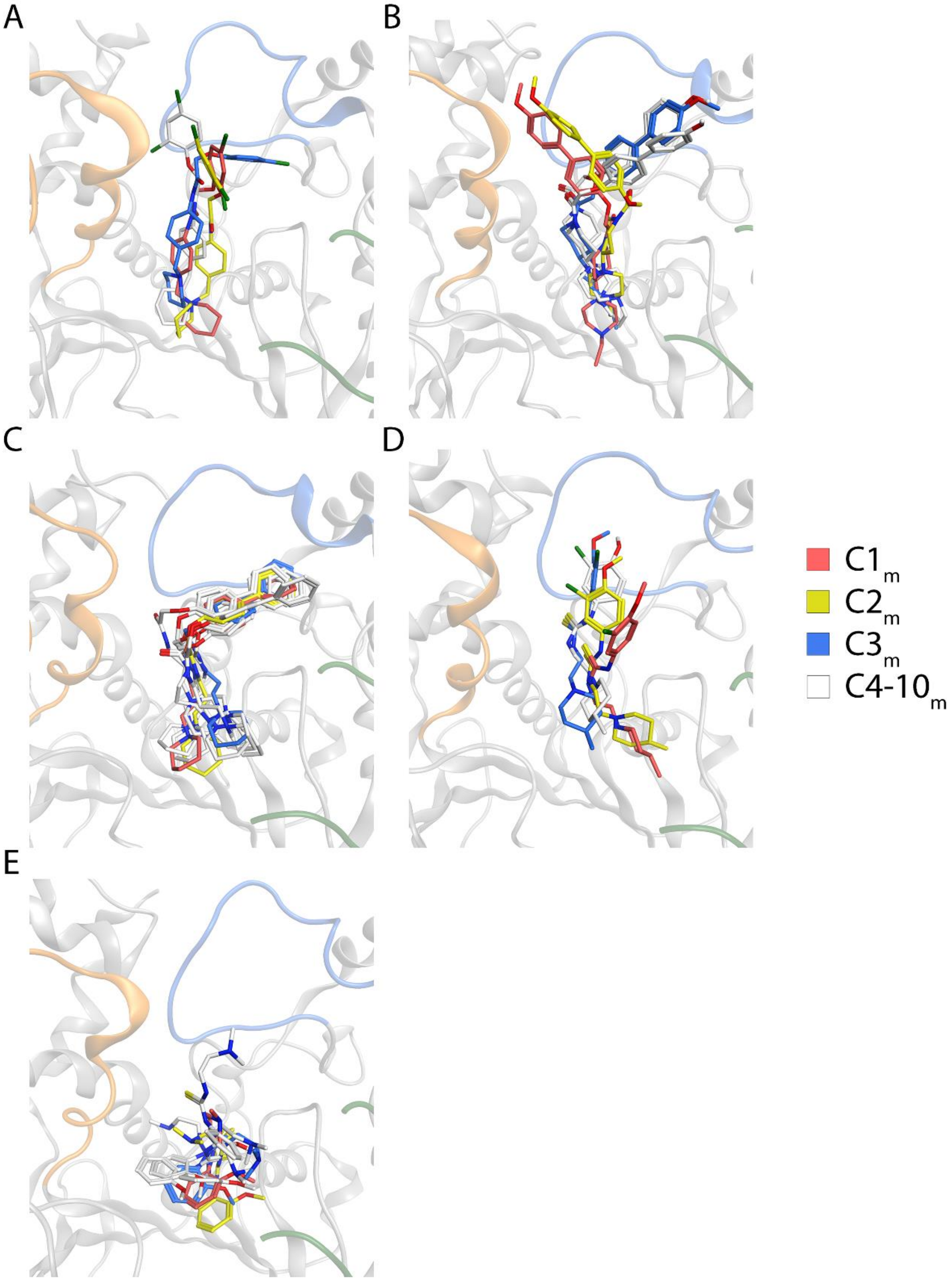

Figure S25. Superposition of conformers of *m*AChE•inhibitors obtained from the cluster analysis performed on protein-ligand interactions using *gmx_clusterByFeatures*. The inhibitors' binding poses of the central conformers of each cluster are shown: the phenoxyacetamide-based inhibitors AL137, AL284, and AL264 (**A-C**), and the *N*-aryl-*N*'-ethyleneaminothioureas AL201 and AL237 (**D-E**). The inhibitors are shown in different colors depending on which cluster it belongs to. The protein ribbons shown in the background are the central conformers of the largest clusters, with the Ω loop in green, loop 1 in blue. and α-helix/loop 2 in orange, respectively. Parts of the Ω loop is removed to better display the inhibitor. Oxygen-, nitrogen-, sulphur- and chlorine atoms are colored in red, blue, yellow, and green, respectively.

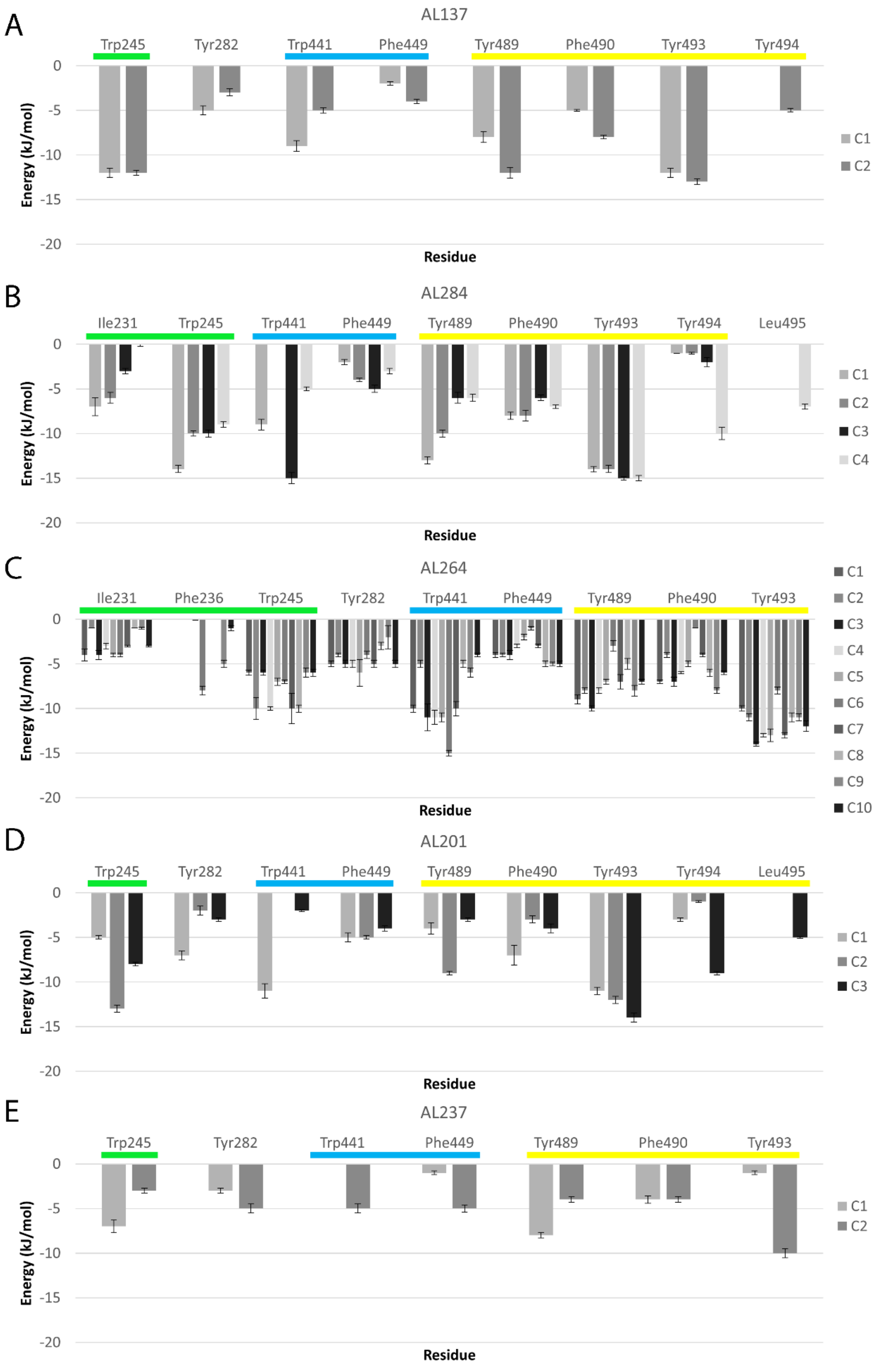

Figure S26. Mean binding energy contributions of interactions between *Ag*AChE1 and inhibitors for each cluster calculated using MMPBSA.[2] **A)** *Ag*AChE1•AL137, **B)** *Ag*AChE1•AL284, **C)** *Ag*AChE1•AL264, **D)** *Ag*AChE1•AL201, and **E)** *Ag*AChE1•AL237. Residues that are part of the three loops are marked with green, cyan, and yellow lines for the Ω loop, loop 1, and α-helix/loop 2, respectively.

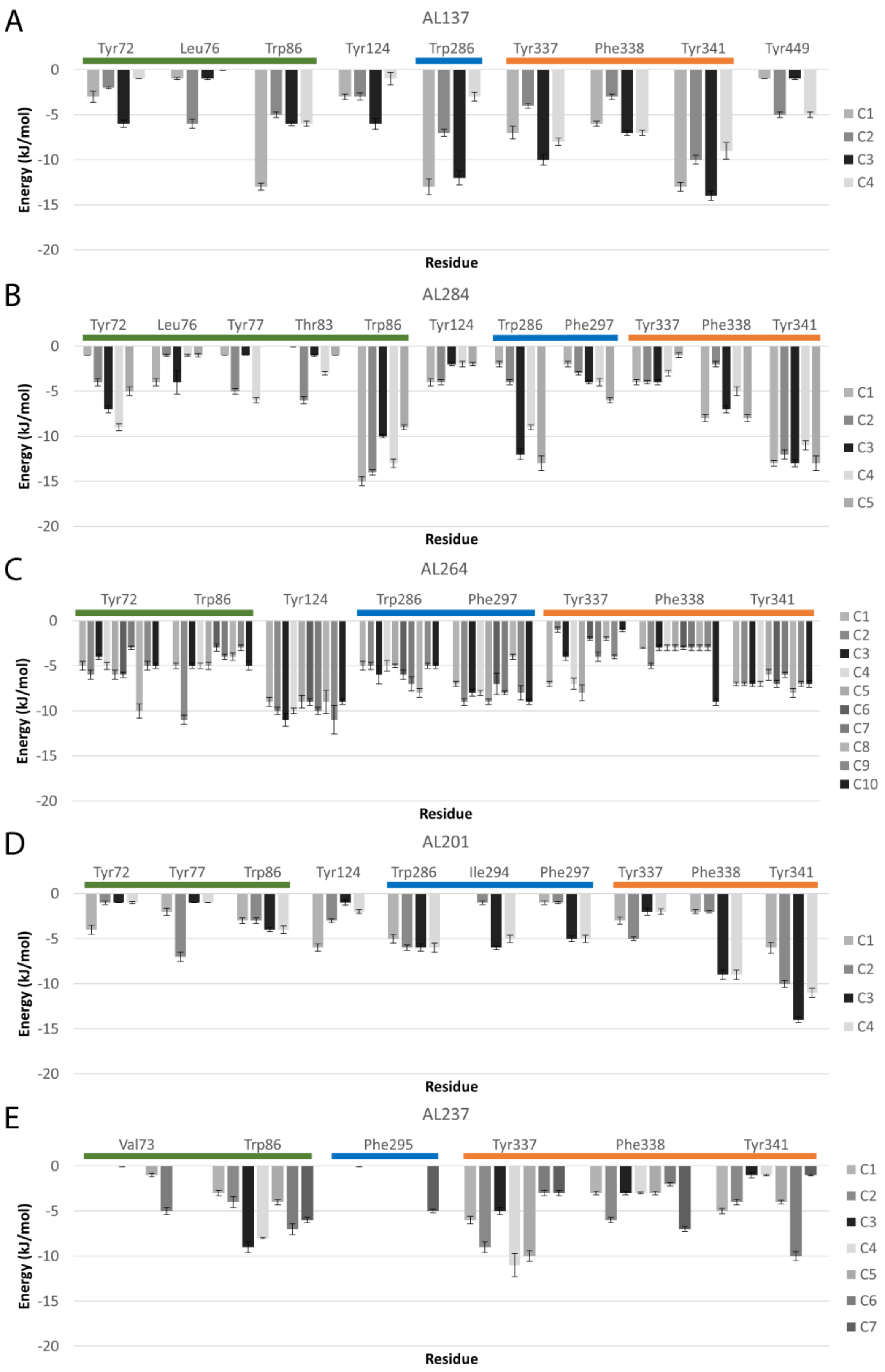

Figure S27. Mean binding energy contributions of interactions between *m*AChE and inhibitors for each cluster calculated using MMPBSA. **A)** *m*AChE•AL137, **B)** *m*AChE•AL284, **C)** *m*AChE•AL264, **D)** *m*AChE•AL201, and **E)** *m*AChE•AL237. Residues that are part of the three loops are marked with dark green, blue, and orange lines for the Ω loop, loop 1, and α-helix/loop 2, respectively.

References

(1) https://gmx-clusterbyfeatures.readthedocs.io/en/latest/.
(2) Kumari, R.; Kumar, R.; Lynn, A. g_mmpbsa—A GROMACS tool for high-throughput MM-PBSA calculations. *J. Chem. Inf. Model.* **2014**, *54* (7), 1951-1962. DOI: 10.1021/ci500020m.
